# Switching from Insulin Degludec plus Dipeptidyl Peptidase-4 Inhibitor to Insulin Degludec/Liraglutide Improves Glycemic Variability in Patients with Type 2 Diabetes: A Preliminary Prospective Observation Study

**DOI:** 10.1155/2022/5603864

**Published:** 2022-01-19

**Authors:** Yuki Oe, Hiroshi Nomoto, Akinobu Nakamura, Saki Kuwabara, Yuka Takahashi, Ayano Yasui, Rimi Izumihara, Aika Miya, Hiraku Kameda, Kyu Yong Cho, Tatsuya Atsumi, Hideaki Miyoshi

**Affiliations:** ^1^Department of Rheumatology, Endocrinology and Nephrology, Faculty of Medicine and Graduate School of Medicine, Hokkaido University, Sapporo, Hokkaido, Japan; ^2^Clinical Research and Medical Innovation Center, Hokkaido University Hospital, Sapporo, Hokkaido, Japan; ^3^Division of Diabetes and Obesity, Faculty of Medicine and Graduate School of Medicine, Hokkaido University, Sapporo, Hokkaido, Japan

## Abstract

Incretins reduce glycemic variability (GV) in patients with type 2 diabetes, but it is unknown whether switching from a combination of basal insulin and a DPP-4 inhibitor to insulin degludec/liraglutide (IDegLira) improves GV. We performed an exploratory prospective observational study to compare the effect of IDegLira and the combination on GV. We recruited hospitalized patients with type 2 diabetes who had stable glycemic control with insulin degludec (≤16 units/day) and taking a DPP-4 inhibitor. GV was analyzed using continuous glucose monitoring (CGM) before and after switching the medication to IDegLira. The principal endpoint was the change in mean amplitude of glycemic excursions (MAGE). Other indices of GV and CGM parameters were analyzed as the secondary endpoints. Fifteen participants were enrolled and 12 completed the study. In these participants, the DPP-4 inhibitor and insulin degludec were discontinued, and the equivalent dose of IDegLira was commenced. Switching to IDegLira significantly improved MAGE from 74.9 (60.3, 97.7) mg/dL to 64.8 (52.0, 78.2) mg/dL (*P* < 0.05), as well as other indices of GV and 24-hour mean blood glucose concentration. Analysis of the ambulatory glucose profile showed marked reductions in postprandial glucose concentration. Nocturnal glucose concentration was similar under the two treatment regimens. IDegLira improved GV as well as the mean and the postprandial glucose concentration by switching from insulin degludec plus DPP-4 inhibitor combination. IDegLira might be beneficial for patients being treated with low-dose basal insulin.

## 1. Introduction

One of the important goals of the treatment of diabetes mellitus is to reduce the incidences of diabetic complications and mortality by improving glycemic control. Various indices have been used to assess glycemic control, and one of these, glycemic variability (GV), has been shown to contribute to the development of atherosclerosis and dementia through an exacerbation of vascular endothelial dysfunction [1]. Although glycated hemoglobin (HbA1c) is frequently used to evaluate glycemic control in clinical practice, it is now thought that stable glycemic control, with minimal GV, is also required for the prevention of diabetic cardiovascular complications in patients with type 2 diabetes (T2DM) [2]. Therefore, the importance of therapies associated with low GV has been advocated, and a number of studies have made use of continuous glucose monitoring (CGM) devices to assess this [3–5].

Recently, injectable fixed-ratio combination formulations of insulin degludec and the glucagon-like peptide-1 receptor agonist (GLP-1RA) liraglutide (IDegLira) have been available. Although insulin treatment reduces fasting plasma glucose (FPG) and HbA1c effectively, it also increases the risks of hypoglycemia and weight gain [6]. On the other hand, GLP-1RAs reduce postprandial glucose, but can have adverse gastrointestinal effects [7]. IDegLira is expected to reduce these risks by decreasing required amount of insulin and GLP-1RA for adequate glycemic control [8].

Even in T2DM patients undergoing insulin therapy, an oral hypoglycemic agent is also commonly administered, and dipeptidyl peptidase-4 (DPP-4) inhibitors are the most frequently used partner for insulin therapy in daily clinical practice in Japan [9]. DPP-4 inhibitors are classified as incretin-related drugs, and their therapeutic mechanism is similar to GLP-1RAs. In previous studies, GLP-1RAs were shown to be more potent to control blood glucose than DPP-4 inhibitors, but the comparisons were performed using high doses of GLP-1RAs; therefore, the effect of low doses of GLP-1RAs on glycemic control, compared to those of DPP-4 inhibitors, remains to be determined [10]. The DUAL II Japan study compared the effect of IDegLira with basal insulin therapy in a Japanese population. It showed IDegLira improved glycemic control more effectively than insulin degludec plus an oral hypoglycemic agent included metformin, but no DPP-4 inhibitor [8]. However, importantly, it is unclear whether IDegLira would also have a superior hypoglycemic effect to the use of basal insulin plus a DPP-4 inhibitor. Furthermore, such comparisons have not yet been reported with respect to GV.

In the present study, we aimed to determine the effect on GV of switching patients with T2DM from treatment with a combination of insulin degludec and a DPP-4 inhibitor to IDegLira using ambulatory CGM. In contrast to previous clinical trials, we evaluated the efficacy of IDegLira at relatively low doses and the participants were restricted to the patients on a combination therapy that included a DPP-4 inhibitor.

## 2. Materials and Methods

### 2.1. Study Design and Participants

We performed an exploratory, prospective, single-arm, observational study. Japanese patients with T2DM who were hospitalized at Hokkaido University Hospital were recruited between February 2020 and April 2021. The participants were thoroughly informed, aged between 20 and 80 years, and had HbA1c values of ≥6.5%. They were instructed to continue their dietary therapy and had had stable blood glucose concentrations for ≥3 days while regularly administering a DPP-4 inhibitor and insulin degludec. A dose of insulin degludec for inclusion criteria was within 16 units/day considering a maximum dose of IDegLira switching from basal insulin therapy was restricted to 16 dose/day. We enrolled patients for whom it was appropriate to switch from the combination therapy of a DPP-4 inhibitor and insulin degludec to IDegLira to achieve better glycemic control. Patients already treated with weekly or daily GLP-1RAs were excluded. The other exclusion criteria were as follows: (1) a history of hypersensitivity to insulin degludec or liraglutide components, (2) unstable diabetic retinopathy, (3) serious liver or renal disease, (4) pregnancy or potential pregnancy, (5) diabetic ketosis/coma or precoma, (6) serious infection, recent or scheduled surgery, or serious trauma, (7) poor compliance with dietary therapy, (8) extremely poor insulin secretory capacity, and (9) unsuitability for another reason.

**Figure 1:**
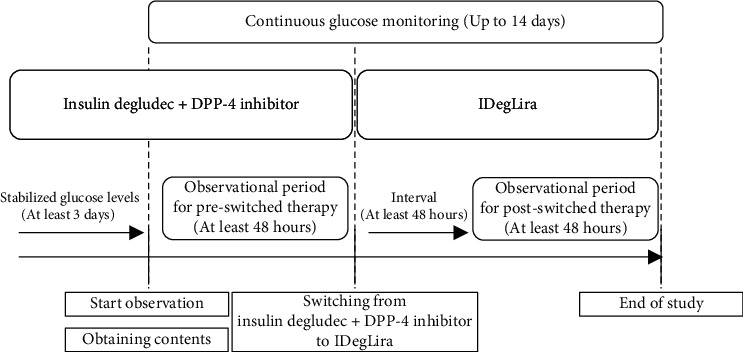
Flowchart for the study. DPP-4i: dipeptidyl peptidase-4 inhibitor; IDegLira: insulin degludec/liraglutide.

The study protocol is illustrated in Figure 1. Before enrolment, the participants had stabilized their fasting blood glucose concentrations by means of therapy that included a combination of insulin degludec and a DPP-4 inhibitor. After obtaining written informed consent from the participants, a CGM device was attached, in accordance with the manufacturer's instructions. Then, the participants were monitored for at least 48 hours while on their original combination therapy, after which they were switched to IDegLira at a dose that was equivalent to their dose of insulin degludec. After a 48-hour transition period, the participants were monitored again for at least 48 hours. After these observation periods, the CGM device was removed, the data were extracted, and the GV indices and CGM parameters for the periods during which the participants were on the combination therapy and IDegLira were compared.

The study was registered with the University Hospital Medical Information Network (UMIN) (registration number UMIN 000039460). It was approved by the Institutional Review Board of Hokkaido University Hospital Clinical Research and Medical Innovation Center (019-0293) and was conducted in accordance with the principles of the Declaration of Helsinki and its amendments. Written informed consent was obtained from all the participants.

### 2.2. Biochemical Analyses and Data Collection

Blood samples were collected after an overnight fast to measure plasma glucose, C-peptide (CPR), HbA1c, and other parameters using standard techniques. Estimated glomerular filtration rate (eGFR) and C-peptide index were calculated using the following formula: eGFR (mL/min/1.73m^2^) = 194 × Cr^−1.094^ (mg/dL) × Age^−0.287^ × 0.739 (if female), according to the Japanese Society of Nephrology criteria, CPI = 100 × fasting CPR (ng/mL)/plasma glucose (mg/dL). A glucagon stimulation test was also performed by intravenously injecting 1 mg glucagon and collecting blood samples for the measurement of plasma glucose and CPR before and 6 min after the injection. C-peptide immunoreactivity after glucagon stimulation (*Δ*CPR) was calculated as the change in this parameter between the two time points. This was used a surrogate for endogenous insulin secretion. Thus, extremely poor insulin secretory capacity was defined as fasting CPR < 0.3 ng/mL and ΔCPR < 0.1 ng/mL. The body mass and height of the participants were measured using a calibrated scale. Body mass index (BMI) was calculated as body mass (kg) divided by height (m^2^). Other data, including the age, sex, diabetes medications, and medical history of the participants, were also collected by the attending physicians.

### 2.3. Continuous Glucose Monitoring

All the participants underwent ambulatory CGM (FreeStyle Libre Pro sensor; Abbott Diabetes Care, Alameda, CA, USA) for up to 14 consecutive days, and we analyzed the CGM data for at least a 48-hour period for each treatment regimen. The following GV parameters were calculated using EasyGV software [11]: the standard deviation of the glucose concentration (SD), mean amplitude of glycemic excursions (MAGE) [12], coefficient of variation (CV) [13], *M* value [14], mean absolute glucose concentration (MAG) [15], continuous overall net glycemic action (CONGA) [16], J-index [17], high blood glucose index (HBGI) [18], mean of the daily difference (MODD) [19], average daily risk range (ADRR) [20], and mean glucose concentration. We also calculated the percentage of the readings and the period of time per day that the blood glucose was within the target range (TIR; 71–180 mg/dL) and the periods of time the glucose concentration was below (TBR; <70 mg/dL) and above (TAR; >180 mg/dL) the target range. The meal times in the hospital started at 08:00, 12:00, and 18:00; we defined glucose concentrations 0-60 minutes before these delivery times as preprandial and 0-180 minutes after these times as postprandial. The nocturnal glucose concentration was defined as the mean glucose concentration between 03:00 and 06:00. We defined severe hypoglycemia as a glucose concentration < 54 mg/dL [21].

### 2.4. Data Analysis

The outcomes were analyzed using CGM data collected during 48 consecutive hours for each treatment. The primary endpoint of the study was the change in MAGE associated with switching treatment. The secondary endpoints were other GV indices calculated using CGM data, as described above, and the mean glucose concentration during each defined time period. Normally distributed data are expressed as mean ± SD and others are expressed as median (interquartile range). Data were analyzed using JMP Pro 14.0.0 (SAS Inc., Cary, NC, USA). For before-and-after comparisons, Student's *t*-test was used to analyze parametric data and Wilcoxon's signed-rank test was used for nonparametric data. All the tests were two-sided, and *P* < 0.05 was considered to represent statistical significance.

## 3. Results

### 3.1. Characteristics of the Participants

**Figure 2:**
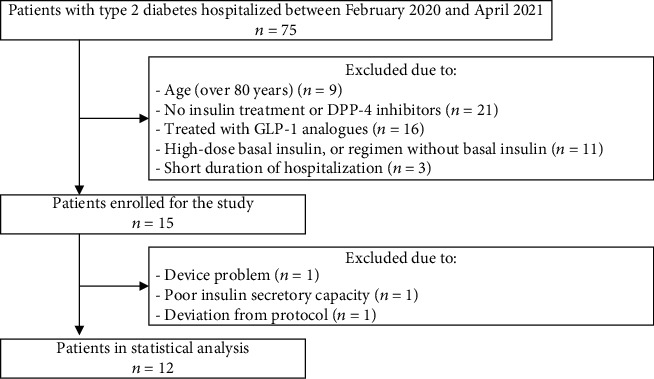
Flow diagram of this study. In total, 75 patients with type 2 diabetes hospitalized during the study periods. Of these, 57 patients were excluded due to exclusion criteria. Fifteen patients were enrolled, and 3 patients dropped out. Finally, 12 patients completed the study. T2DM: type 2 diabetes; DPP-4 inhibitor: dipeptidyl peptidase-4 inhibitor; GLP-1RA: glucagon-like peptide-1 receptor agonist.

A flow diagram of this study is shown in Figure 2. During the study period, 75 patients with T2DM were admitted to our department and were screened for eligibility. Finally, we obtained informed consent from 15 patients; however, three were excluded because of problems with the device, deviation from the protocol, and poor insulin secretory capacity (*n* = 1 each). Therefore, 12 participants completed the study and their data were analyzed. None of the participants adjusted the amount of insulin administered during the first observation period, and all the other drugs, except for DPP-4 inhibitors, were continued during the study periods.  Table 1 shows details of patient characteristics. Patients tended to be obese in Japanese criteria, and no patients were insulin dependent. The number of units of insulin degludec that were administered during the first assessment period was 7 (4.0, 9.5), and the usage of oral hypoglycemic agents other than DPP-4 inhibitors is shown in Table 1. The median dose of liraglutide contained in IDegLira was 0.25 (0.14, 0.34) mg/day. The DPP-4 inhibitors used were linagliptin (5 mg; *n* = 5), vildagliptin (100 mg; *n* = 4), and sitagliptin (50 mg; *n* = 3). Only one of the participants administered bolus insulin therapy in addition to insulin degludec, at a total daily dose of 14 units, and this was not changed during the observation period.

Importantly, we confirmed the stability of the plasma glucose concentration and the condition of the participants by the self-measurement of blood glucose concentration (SMBG) and body mass during preobservation and observation periods. The preobservation period started at least 3 days before the first observation period. No significant differences were found between these two periods with respect to FPG and body mass, which implies that the effects of hospitalization on these parameters were minimal (Supplementary Table (available here)).

### 3.2. Comparison of Glucose Variability during Combination Therapy and IDegLira

**Figure 3:**
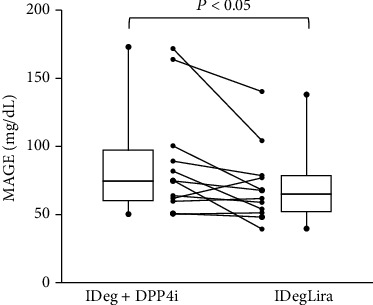
Mean amplitude of glycemic excursions before and after switching to insulin degludec/liraglutide. The figure was constructed for all 12 participants, and the error bars represent the standard deviation of measurements. All data was nonparametric and analyzed by Wilcoxon's signed-rank test. IDeg: insulin degludec; DPP-4i: dipeptidyl peptidase-4 inhibitor; IDegLira: insulin degludec/liraglutide. The error bars represent the standard deviation of measurements.

Table 2 shows a comparison of the CGM parameters during combination therapy and IDegLira. The main endpoint of the study, MAGE, significantly decreased from 74.9 (60.3, 97.7) mg/dL to 64.8 (52.0, 78.2) mg/dL (*P* < 0.05) (Figure 3). Regarding the secondary endpoints, most of the other indices of GV significantly improved (Table 2, Supplementary Figures 1 and 2). The indices of the daily fluctuation in blood glucose concentration, the *M* value and MAG, significantly decreased (*P* < 0.01). The indices of the daily variation in blood glucose concentration, MODD and ADRR, also significantly decreased (*P* < 0.05). Furthermore, the other CGM parameters, especially those that are indicative of the risk associated with hyperglycemia, the J-index and HBGI, also significantly improved (*P* < 0.01). TAR significantly decreased, whereas TIR did not change and TBR increased after switching (*P* < 0.05).

### 3.3. Evaluation of Ambulatory Glucose Profile, according to the Time of Day

**Figure 4:**
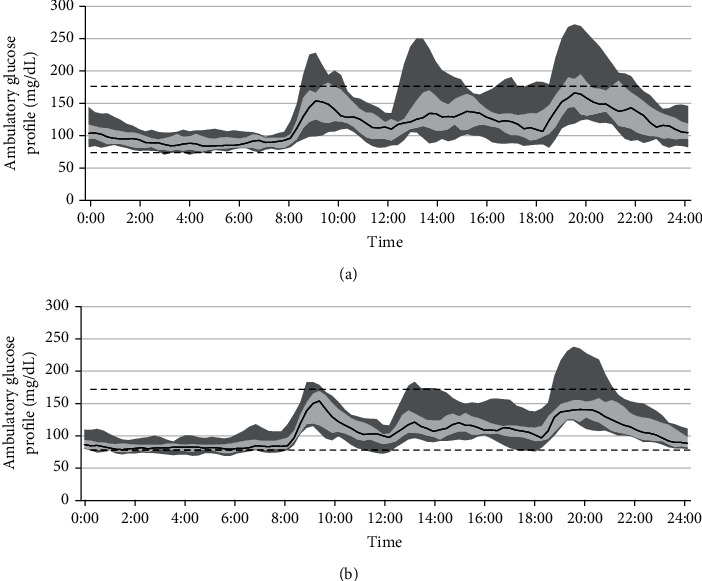
Ambulatory glucose profiles (AGPs). AGPs were constructed for all 12 participants. The black line represents the median glucose concentration, and the bands, which comprised small dots and larger dots, represent the 25th–75th and 10th–90th percentiles, respectively. The dotted line represents the target glucose range. (a) Combination therapy comprising a DPP-4 inhibitor and insulin degludec. (b) IDegLira. IDeg: insulin degludec; DPP-4i: dipeptidyl peptidase-4 inhibitor; IDegLira: insulin degludec/liraglutide.

**Figure 5:**
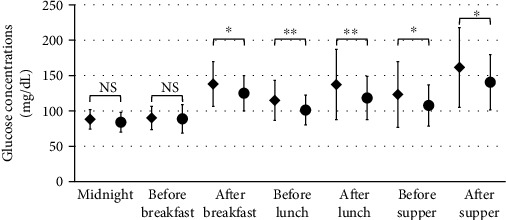
Mean glucose concentrations during the phases of the day. The figure was constructed for all 12 participants, and the error bars represent the standard deviation of measurements. All data was parametric and analyzed by Student's *t*-test. The mean glucose concentrations are shown for the preprandial periods, postprandial periods, and overnight. The preprandial phase was defined as 60 minutes before the delivery of a meal and the postprandial phase as 180 minutes from the start of the meal. The nocturnal phase was defined as 03:00–06:00. Black diamond: insulin degludec plus a dipeptidyl peptidase-4 inhibitor; black circle, insulin degludec/liraglutide. ^∗∗^*P* < 0.01; ^∗^*P* < 0.05; NS: not significant.

We constructed ambulatory glucose profiles (AGPs) and analyzed the glucose fluctuations during each period of the day (Figures 4 and 5). The AGPs include the median glucose concentrations, shown as a line, and GV, shown as bands, corresponding to the 25th–75th and 10th–90th percentiles. The IDegLira treatment was associated with an improvement in the median glucose concentration and narrowing of the 25th–75th and 10th–90th percentiles, especially during the postprandial period (Figure 4). Indeed, the mean postprandial glucose concentration significantly decreased after all the meals, as shown in Figure 5 (*P* < 0.01) and Supplementary Figure 3. Furthermore, the glucose concentrations prior to lunch and dinner also significantly decreased (*P* < 0.05), whereas the nocturnal and morning glucose concentrations did not, which may contribute to the avoidance of nocturnal hypoglycemia. Although the analysis was limited to the preprandial and after-supper data, we confirmed that the SMBG data showed the same trends as the CGM data (Supplementary Figure 4).

### 3.4. Adverse Events

There were no adverse events, including gastrointestinal symptoms associated with the switch to IDegLira. Symptomatic hypoglycemia was not observed in all patients. One participant experienced nocturnal asymptomatic severe hypoglycemia during the first evaluation period, when under treatment with a DPP-4 inhibitor and insulin degludec, but this did not recur after switching to IDegLira, without a change in the insulin dose.

## 4. Discussion

In the present study, we evaluated the effects of IDegLira on GV after switching from a combination of insulin degludec and a DPP-4 inhibitor in hospitalized Japanese patients with T2DM, using CGM. To the best our knowledge, this is the first study to show that IDegLira has beneficial effects in patients that were previously administering a combination of low-dose basal insulin and DPP-4 inhibitors. Our study presents two important findings. First, a small dose of a GLP-1RA, in place of the usual dose of a DPP-4 inhibitor, improved glycemic control, including GV and postprandial hyperglycemia. Second, switching to IDegLira, while maintaining the same basal insulin dose, was sufficient to obtain a superior therapeutic effect without causing undesirable side effects, such as nocturnal hypoglycemia or gastrointestinal symptoms.

In previous phase III trials, IDegLira was more effective on HbA1c reduction than insulin degludec [8, 22]. However, the doses of basal insulin and IDegLira at the end of study were almost 30–40 units/day, being higher than the typical basal insulin dose recorded in a previous study of a Japanese database [23]. Patients on DPP-4 inhibitors were not enrolled in those phase III trials, despite the high prescription rate of these drugs in Japan [9], which hampers understanding of the efficacy of IDegLira in the real world. The present study shows the efficacy of a low dose of IDegLira in patients previously treated with a DPP-4 inhibitor.

Poor GV contributes to the development of diabetes-related atherosclerotic complications, resulting in cardiovascular diseases and dementia [1]. Although HbA1c has been shown to be associated with diabetic complications, some clinical trials have shown that intensive glycemic control assessed using HbA1c does not always delay the onset of cardiovascular disease in patients with T2DM [24]. The results of recent clinical studies have suggested that GV is more strongly associated with cardiovascular events than HbA1c [25]. In the present study, several indices of GV were analyzed. Since they were immediately calculated by casual glucose concentration, their changes would reflect real-time efficacy of new therapeutic strategies. We showed IDegLira improved a number of indices of GV including MAGE, the main endpoint of this study, versus the tested combination therapy, which may help prevent the progression of atherosclerosis.

The FreeStyle Libre Pro sensor, which was the CGM device used in the present study, showed low mean glucose concentrations and had low accuracy in the hypoglycemic range and the measurable limit was 14 days [26]. In the present study, TAR decreased but TBR increased, and only CV did not change even though other various indices improved dramatically. A treatment regimen including GLP-1RAs is associated with a low risk of hypoglycemia [27], and the measurement for a part of GV indices such as CV requires relatively long period [28]. Therefore, the change in TBR recorded may have been affected by the specific CGM device used, and short-term observation in this study might affect the result of CV. There is a concern that the accuracy of the CGM device is variable, especially just after attachment. To investigate this possibility, we assessed the glucose fluctuations using SMBG four times a day and found similar trends to those identified using CGM. However, it must be borne in mind that hypoglycemic episodes can be observed when a GLP-1RA is added to insulin or an insulin secretagogue [29].

Figures 4 and 5 show the beneficial effects of IDegLira on GV. IDegLira reduced the mean glucose concentration and had a marked effect to suppress postprandial hyperglycemia. Insulin degludec, a basal insulin preparation, also reduces mean glucose concentration and FPG [30], but effects on the postprandial glucose concentration have a greater impact on glycemic control when FPG is effectively controlled [31]. In a previous study, it was shown that postprandial hyperglycemia was strongly associated with the development of cardiovascular diseases [32]. In the present study, the nocturnal glucose concentration was not affected by switching therapy and FPG was well controlled, even during the preobservation period. Therefore, IDegLira may be more effective at reducing postprandial hyperglycemia than conventional treatments that include insulin degludec.

Liraglutide has been shown to reduce the incidence of cardiovascular events, albeit at a high dose [33]. A previous clinical trial showed that it controls glycemia even at a relatively low dose [34], but the licensed dose is ≥0.3 mg/day and its effectiveness at lower doses has not yet been fully assessed. Of note, the median dose of liraglutide administered as part of IDegLira was only 0.25 mg/day in the present study, less than the conventional dose. Therefore, liraglutide may be more effective than a regular dose of a DPP-4 inhibitor, even at a low dose. Both DPP-4 inhibitors and GLP-1RAs are categorized as incretin-related drugs. DPP-4 inhibitors prevent the inactivation of native GLP-1, and although they have the advantage of being administered orally, their effects on HbA1c are generally lesser extent than those of GLP-1RAs [35]. Some previous clinical trials have directly compared GLP-1RAs to DPP-4 inhibitors, but the doses of GLP-1RAs used were relatively high [10]. It is unclear how the dose of a GLP-1RA compares to the dose of a DPP-4 inhibitor. Importantly, even in previous trials of IDegLira, patients who were taking a DPP-4 inhibitor were not enrolled [8]. Considering patients with T2DM tended to be treated with DPP-4 inhibitors [9], our results may provide useful treatment strategy in a real-world setting.

The present study had several limitations. First, it was a single-arm observational study and the sample size was small. We waited until the participants' glucose concentrations had stabilized before starting the observations (as shown in the Supplementary Table) to minimize the limitation of lack of a control group. A larger, two-arm study would have been desirable. Second, the observation period was a maximum of 2 weeks because of the period of operation of the CGM sensor; therefore, the durability of the effects and the adverse events associated with longer-term use should be studied in the future. Third, we could not fully avoid carry-over effects after switching. We set a washout period of at least 48 hours, enough for the DPP-4 inhibitors to be metabolized and excreted, according to their half-lives. Fourth, the participants were limited to hospitalized patients, and the dietary therapy administered in the hospital may also have affected their glucose concentrations and GV. Lastly, none of the participants were taking a sulfonylurea at the start of the present study. Considering that a combination of an incretin and a sulfonylurea is efficacious at promoting insulin secretion, the present findings should be confirmed in patients who are taking a sulfonylurea in a future study.

## 5. Conclusions

IDegLira was associated with better glycemic control than combination therapy comprising a DPP-4 inhibitor and insulin degludec. IDegLira improved GV and had a therapeutic effect even at a low dose. We expect that IDegLira will help to reduce cardiovascular events in the long term.

## Figures and Tables

**Table 1:** Participant characteristics.

Variables	Total (*n* = 12)
Age (years)	67.5 (53.5, 69.8)
Female sex (*n*)	7
BMI (kg/m^2^)	26.8 (23.7, 27.9)
Diabetes duration (years)	3.5 (0.5, 15.8)
FPG (mg/dL)	117.0 (102.8, 179.5)
HbA1c (%)	9.6 ± 2.0
CPR (ng/mL)	1.97 ± 1.2
CPI (ng/mL per mg/dL)	1.4 ± 0.8
*Δ*CPR (ng/mL)	0.9 ± 0.8
UACR (mg/gCr)	11.9 (4.2, 41.8)
eGFR (mL/min/1.73m^2^)	70.1 ± 16.1
IDeg (unit/day)	7 (4.0, 9.5)
IDegLira after switching (dose/day)	7 (4.0, 9.5)
The proportion of oral hypoglycemic agent	
Metformin (%)	8 (66.7)
Alpha glucosidase inhibitors (%)	1 (8.3)
Glinides (%)	1 (8.3)
SGLT2 inhibitors (%)	3 (25.0)
Bolus insulin (%)	1 (8.3)
Total daily dose (units)	14

Values are expressed as mean ± SD or median (interquartile range). BMI: body mass index; FPG: fasting plasma glucose; HbA1c: glycated hemoglobin; CPR: C-peptide; CPI: C-peptide index; *Δ*CPR: C-peptide immunoreactivity after glucagon stimulation; UACR: urine albumin to creatinine ratio; eGFR: estimated glomerular filtration rate; IDeg: insulin degludec before switching; IDegLira: insulin degludec/liraglutide after switching; SGLT2: sodium-glucose cotransporter 2.

**Table 2:** Parameters describing glucose variability in participants being treated with combination therapy or IDegLira.

	IDeg+DPP-4i	IDegLira	*P* value
MAGE (mg/dL)	74.9 (60.3, 97.7)	64.8 (52.0, 78.2)	<0.05^†^
*M* value (mg/dL)	1975.2 ± 457.0	1748.9 ± 364.8	<0.01
MAG (mg/dL)	26.1 ± 8.1	22.1 ± 6.7	<0.01
MODD (mg/dL)	19.9 ± 7.0	15.0 ± 4.4	<0.05
ADRR (mg/dL)	431.5 ± 70.1	404.8 ± 61.2	<0.05
J-index	6776.9 (5553.8, 10510.1)	4984.3 (4412.7, 8123.0)	<0.01^†^
CONGA (mg/dL)	108.7 ± 26.6	96.2 ± 19.6	<0.01
HBGI	311.9 ± 47.6	288.7 ± 39.3	<0.01
24 h mean glucose (mg/dL)	122.3 ± 28.9	108.4 ± 21.5	<0.01
CV (%)	24.6 ± 7.7	29.3 ± 22.4	0.54
SD (mg/dL)	28.2 (18.8, 36.6)	21.9 (18.0, 23.7)	<0.05^†^
TBR (%)	0.5 (0, 1.4)	2.6 (0, 5.5)	<0.05^†^
TIR (%)	94.3 (69.0, 99.0)	94.5 (85.4, 97.4)	0.56^†^
TAR (%)	2.3 (0, 20.8)	0 (0, 5.2)	<0.05^†^

Values are expressed as mean ± SD or median (interquartile range). *P* value of IDeg+DPP-4i vs. IDegLira. ^†^Wilcoxon signed-rank test was applied to the factors: MAGE, J-index, SD, TBR, TIR, and TAR. MAGE: mean amplitude of glycemic excursions; MAG: mean absolute glucose; MODD: mean of daily difference; ADRR: average daily risk range; CONGA: continuous overall net glycemic action; HGBI: high blood glucose index; CV: coefficient of variation; SD: standard deviation; TBR: time below target glucose range; TIR: the target glucose range; TAR: time above target glucose range.

## Data Availability

The datasets used and/or analyzed during the current study are available from the corresponding author on reasonable request.

## Supplementary Materials

Supplementary 1 Supplementary Table: confirmation of the stability of participant glucose concentrations before the study.

Supplementary 2 Supplementary Figure 1: individual changes in variables related to the daily fluctuation in blood glucose concentration. The figures were constructed for all 12 participants, and the error bars represent the standard deviation of measurements. Student's *t*-test was used to analyze data: (a) *M* value and (b) mean absolute glucose. White circles: mean ± SD. IDeg: insulin degludec; DPP-4i: dipeptidyl peptidase-4 inhibitor; IDegLira: insulin degludec/liraglutide.

Supplementary 3 Supplementary Figure 2: individual changes in the daily difference variation in blood glucose concentration. The figures were constructed for all 12 participants, and the error bars represent the standard deviation of measurements. Student's *t*-test was used to analyze data: (a) mean of the daily difference and (b) average daily risk range. White circles: mean ± SD. IDeg: insulin degludec; DPP-4i: dipeptidyl peptidase-4 inhibitor; IDegLira: insulin degludec/liraglutide.

Supplementary 4 Supplementary Figure 3: individual sets of continuous glucose monitoring data before and after changing the treatment. The panels show the data for all 12 participants. The black lines represent the median glucose concentrations during the observation period before and after switching the treatment. IDeg: insulin degludec; DPP-4i: dipeptidyl peptidase-4 inhibitor; IDegLira: insulin degludec/liraglutide.

Supplementary 5 Supplementary Figure 4: self-measured glucose concentrations during each phase of the day. Mean self-measured glucose concentrations during each phase of the day. The data are for all 12 participants, and the error bars represent the standard deviation of the measurements. All the datasets were normally distributed and analyzed using Student's *t*-test. The mean preprandial and after-supper glucose concentrations are shown. Black diamond: insulin degludec plus a dipeptidyl peptidase-4 inhibitor; black circle: insulin degludec/liraglutide. ^∗^*P* < 0.05; NS: not significant.
