# Supplementary material for: Switching from Insulin Degludec plus Dipeptidyl Peptidase-4 Inhibitor to Insulin Degludec/Liraglutide Improves Glycemic Variability in Patients with Type 2 Diabetes: A Preliminary Prospective Observation Study
Source: J Diabetes Res. 2022 Jan 19;2022:5603864. doi: 10.1155/2022/5603864 (PMC8793345; doi:10.1155/2022/5603864)
Supplement: Supplementary 1 — Supplementary Table: confirmation of the stability of participant glucose concentrations before the study. [file 5603864.f1.docx]

Supplementary Table. Confirmation of glucose stability before evaluation.

|  | pre-evaluation | first-evaluation | *P* value |
| --- | --- | --- | --- |
| FPG (mg/dL) | 116.8 ± 12.4 | 115.1 ± 16.0 | 0.61 |
| BW (kg) | 67.4 (58.8, 75.8) | 67.5 (58.8, 75.3) | 0.41† |

Values are expressed as mean ± SD or median (interquartile range). *P* value of pre-evaluation vs evaluation of pre-switching period. †Wilcoxon signed-rank test was applied to BW. FPG, fasting plasma glucose; BW, body weight.
