# Supplementary figures and images for: Switching from Insulin Degludec plus Dipeptidyl Peptidase-4 Inhibitor to Insulin Degludec/Liraglutide Improves Glycemic Variability in Patients with Type 2 Diabetes: A Preliminary Prospective Observation Study
Source: J Diabetes Res. 2022 Jan 19;2022:5603864. doi: 10.1155/2022/5603864 (PMC8793345; doi:10.1155/2022/5603864)

## Slide 1
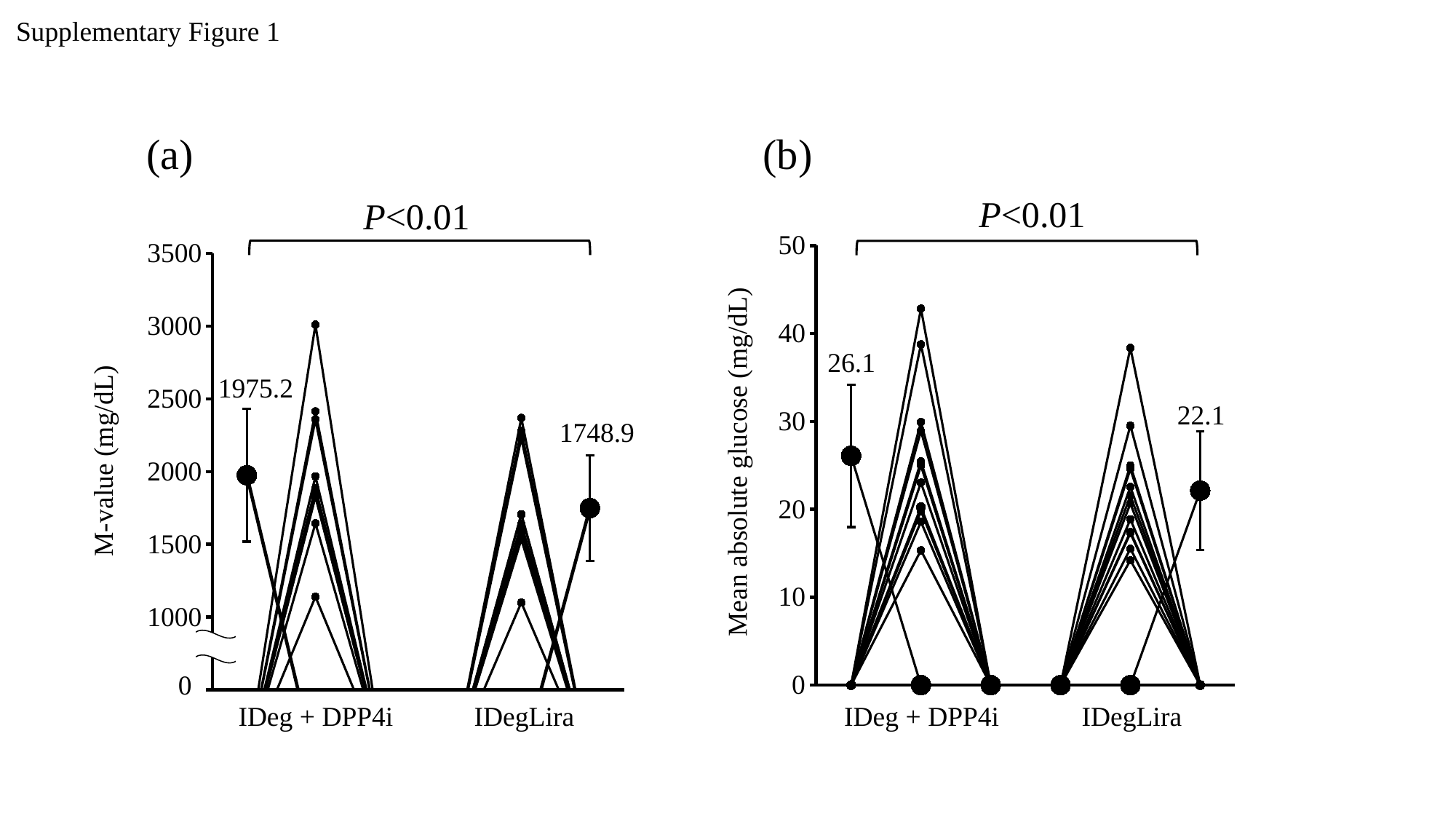

Supplement: Supplementary 2 — Supplementary Figure 1: individual changes in variables related to the daily fluctuation in blood glucose concentration. The figures were constructed for all 12 participants, and the error bars represent the standard deviation of measurements. Student's t-test was used to analyze data: (a) M value and (b) mean absolute glucose. White circles: mean ± SD. IDeg: insulin degludec; DPP-4i: dipeptidyl peptidase-4 inhibitor; IDegLira: insulin degludec/liraglutide. [file 5603864.f2.pptx]

## Slide 1
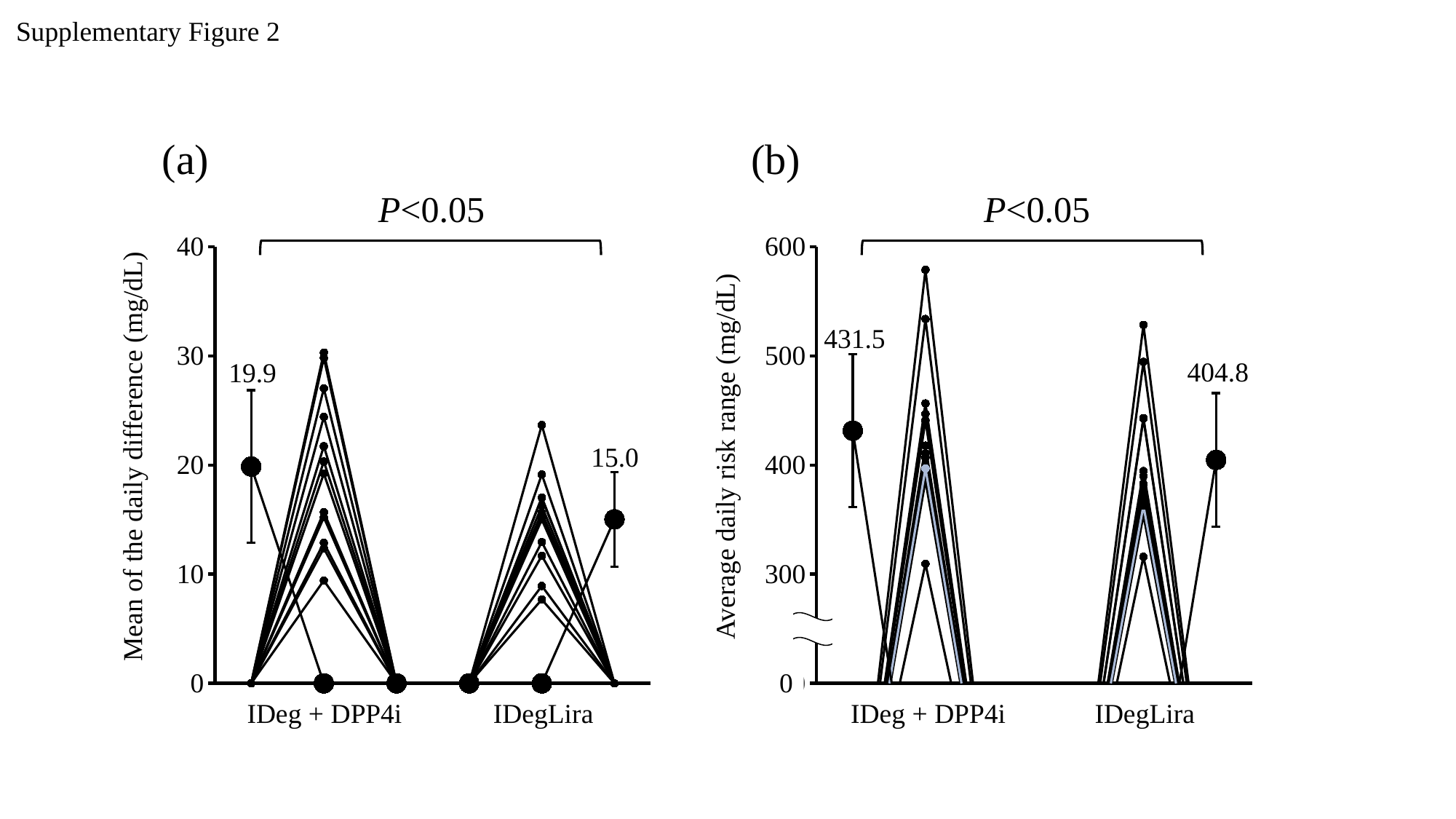

Supplement: Supplementary 3 — Supplementary Figure 2: individual changes in the daily difference variation in blood glucose concentration. The figures were constructed for all 12 participants, and the error bars represent the standard deviation of measurements. Student's t-test was used to analyze data: (a) mean of the daily difference and (b) average daily risk range. White circles: mean ± SD. IDeg: insulin degludec; DPP-4i: dipeptidyl peptidase-4 inhibitor; IDegLira: insulin degludec/liraglutide. [file 5603864.f3.pptx]
