# Supplementary material for: Switching from Insulin Degludec plus Dipeptidyl Peptidase-4 Inhibitor to Insulin Degludec/Liraglutide Improves Glycemic Variability in Patients with Type 2 Diabetes: A Preliminary Prospective Observation Study
Source: J Diabetes Res. 2022 Jan 19;2022:5603864. doi: 10.1155/2022/5603864 (PMC8793345; doi:10.1155/2022/5603864)
Supplement: Supplementary 4 — Supplementary Figure 3: individual sets of continuous glucose monitoring data before and after changing the treatment. The panels show the data for all 12 participants. The black lines represent the median glucose concentrations during the observation period before and after switching the treatment. IDeg: insulin degludec; DPP-4i: dipeptidyl peptidase-4 inhibitor; IDegLira: insulin degludec/liraglutide. [file 5603864.f4.pptx]

## Slide 1
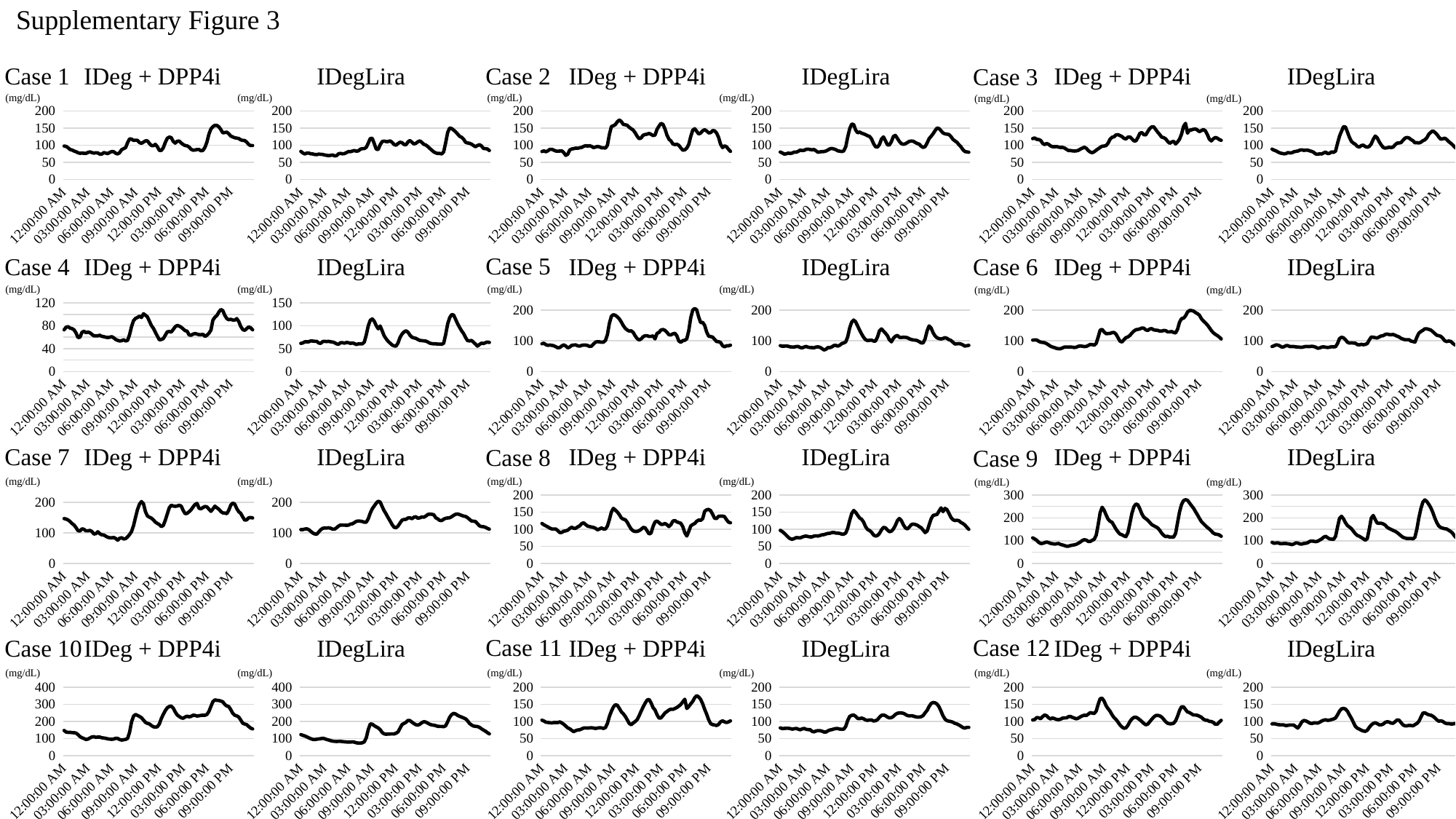

Supplementary Figure 3
Case 2
Case 1
IDeg + DPP4i
IDegLira
IDeg + DPP4i
IDegLira
IDeg + DPP4i
IDegLira
IDeg + DPP4i
IDegLira
IDeg + DPP4i
IDegLira
IDeg + DPP4i
IDegLira
IDeg + DPP4i
IDegLira
IDeg + DPP4i
IDegLira
IDeg + DPP4i
IDegLira
IDeg + DPP4i
IDegLira
IDeg + DPP4i
IDegLira
IDeg + DPP4i
IDegLira
Case 3
(mg/dL)
(mg/dL)
(mg/dL)
(mg/dL)
(mg/dL)
(mg/dL)
### Chart
| Category | |
|---|---|
| 0 | 97.5 |
| 1.0416666666666666E-2 | 96.5 |
| 2.0833333333333301E-2 | 92.5 |
| 3.125E-2 | 87.5 |
| 4.1666666666666699E-2 | 86.0 |
| 5.2083333333333301E-2 | 83.0 |
| 6.25E-2 | 81.0 |
| 7.2916666666666699E-2 | 78.0 |
| 8.3333333333333301E-2 | 76.5 |
| 9.375E-2 | 77.0 |
| 0.104166666666667 | 76.5 |
| 0.114583333333333 | 76.0 |
| 0.125 | 79.0 |
| 0.13541666666666699 | 80.5 |
| 0.14583333333333301 | 78.5 |
| 0.15625 | 77.0 |
| 0.16666666666666699 | 78.0 |
| 0.17708333333333301 | 77.5 |
| 0.1875 | 74.0 |
| 0.19791666666666699 | 74.0 |
| 0.20833333333333301 | 78.5 |
| 0.21875 | 77.0 |
| 0.22916666666666699 | 75.5 |
| 0.23958333333333301 | 79.0 |
| 0.25 | 81.5 |
| 0.26041666666666702 | 81.0 |
| 0.27083333333333298 | 76.5 |
| 0.28125 | 74.5 |
| 0.29166666666666702 | 78.5 |
| 0.30208333333333298 | 87.0 |
| 0.3125 | 90.0 |
| 0.32291666666666702 | 93.0 |
| 0.33333333333333298 | 107.5 |
| 0.34375 | 118.5 |
| 0.35416666666666702 | 117.5 |
| 0.36458333333333298 | 114.0 |
| 0.375 | 114.5 |
| 0.38541666666666702 | 114.0 |
| 0.39583333333333298 | 107.5 |
| 0.40625 | 105.5 |
| 0.41666666666666702 | 109.0 |
| 0.42708333333333298 | 112.5 |
| 0.4375 | 112.5 |
| 0.44791666666666702 | 105.0 |
| 0.45833333333333298 | 99.0 |
| 0.46875 | 98.5 |
| 0.47916666666666702 | 102.5 |
| 0.48958333333333298 | 95.5 |
| 0.5 | 85.0 |
| 0.51041666666666696 | 84.5 |
| 0.52083333333333304 | 92.5 |
| 0.53125 | 108.0 |
| 0.54166666666666696 | 120.0 |
| 0.55208333333333304 | 123.5 |
| 0.5625 | 122.0 |
| 0.57291666666666696 | 111.0 |
| 0.58333333333333304 | 106.0 |
| 0.59375 | 111.5 |
| 0.60416666666666696 | 112.0 |
| 0.61458333333333304 | 106.5 |
| 0.625 | 102.0 |
| 0.63541666666666696 | 99.0 |
| 0.64583333333333304 | 98.0 |
| 0.65625 | 94.5 |
| 0.66666666666666696 | 88.0 |
| 0.67708333333333304 | 85.5 |
| 0.6875 | 86.0 |
| 0.69791666666666696 | 87.5 |
| 0.70833333333333304 | 87.5 |
| 0.71875 | 83.5 |
| 0.72916666666666696 | 85.0 |
| 0.73958333333333304 | 94.0 |
| 0.75 | 109.5 |
| 0.76041666666666696 | 132.5 |
| 0.77083333333333304 | 147.5 |
| 0.78125 | 154.0 |
| 0.79166666666666696 | 158.0 |
| 0.80208333333333304 | 157.5 |
| 0.8125 | 153.5 |
| 0.82291666666666696 | 145.5 |
| 0.83333333333333304 | 136.0 |
| 0.84375 | 137.0 |
| 0.85416666666666696 | 138.0 |
| 0.86458333333333304 | 133.0 |
| 0.875 | 126.5 |
| 0.88541666666666696 | 124.0 |
| 0.89583333333333304 | 121.5 |
| 0.90625 | 120.5 |
| 0.91666666666666696 | 119.5 |
| 0.92708333333333304 | 115.5 |
| 0.9375 | 114.0 |
| 0.94791666666666696 | 113.5 |
| 0.95833333333333304 | 109.0 |
| 0.96875 | 102.0 |
| 0.97916666666666696 | 99.0 |
| 0.98958333333333304 | 99.0 |
### Chart
| Category | |
|---|---|
| 0 | 82.0 |
| 1.0416666666666666E-2 | 77.5 |
| 2.0833333333333301E-2 | 74.5 |
| 3.125E-2 | 77.0 |
| 4.1666666666666699E-2 | 77.0 |
| 5.2083333333333301E-2 | 75.0 |
| 6.25E-2 | 74.5 |
| 7.2916666666666699E-2 | 73.0 |
| 8.3333333333333301E-2 | 72.5 |
| 9.375E-2 | 74.0 |
| 0.104166666666667 | 73.5 |
| 0.114583333333333 | 73.0 |
| 0.125 | 71.5 |
| 0.13541666666666699 | 70.5 |
| 0.14583333333333301 | 69.5 |
| 0.15625 | 70.5 |
| 0.16666666666666699 | 71.5 |
| 0.17708333333333301 | 69.0 |
| 0.1875 | 69.5 |
| 0.19791666666666699 | 75.0 |
| 0.20833333333333301 | 76.0 |
| 0.21875 | 75.0 |
| 0.22916666666666699 | 75.5 |
| 0.23958333333333301 | 78.5 |
| 0.25 | 81.5 |
| 0.26041666666666702 | 81.0 |
| 0.27083333333333298 | 83.0 |
| 0.28125 | 84.5 |
| 0.29166666666666702 | 82.5 |
| 0.30208333333333298 | 83.5 |
| 0.3125 | 88.5 |
| 0.32291666666666702 | 90.0 |
| 0.33333333333333298 | 90.0 |
| 0.34375 | 94.0 |
| 0.35416666666666702 | 106.0 |
| 0.36458333333333298 | 119.5 |
| 0.375 | 120.0 |
| 0.38541666666666702 | 105.0 |
| 0.39583333333333298 | 88.5 |
| 0.40625 | 87.5 |
| 0.41666666666666702 | 101.5 |
| 0.42708333333333298 | 110.5 |
| 0.4375 | 112.0 |
| 0.44791666666666702 | 110.5 |
| 0.45833333333333298 | 110.5 |
| 0.46875 | 112.5 |
| 0.47916666666666702 | 108.5 |
| 0.48958333333333298 | 102.0 |
| 0.5 | 100.0 |
| 0.51041666666666696 | 105.0 |
| 0.52083333333333304 | 108.5 |
| 0.53125 | 107.5 |
| 0.54166666666666696 | 102.0 |
| 0.55208333333333304 | 101.0 |
| 0.5625 | 109.5 |
| 0.57291666666666696 | 113.5 |
| 0.58333333333333304 | 108.5 |
| 0.59375 | 103.5 |
| 0.60416666666666696 | 105.5 |
| 0.61458333333333304 | 110.0 |
| 0.625 | 112.5 |
| 0.63541666666666696 | 108.0 |
| 0.64583333333333304 | 102.5 |
| 0.65625 | 100.5 |
| 0.66666666666666696 | 96.0 |
| 0.67708333333333304 | 90.5 |
| 0.6875 | 85.5 |
| 0.69791666666666696 | 80.5 |
| 0.70833333333333304 | 77.5 |
| 0.71875 | 76.0 |
| 0.72916666666666696 | 76.0 |
| 0.73958333333333304 | 74.5 |
| 0.75 | 81.0 |
| 0.76041666666666696 | 107.5 |
| 0.77083333333333304 | 137.0 |
| 0.78125 | 150.0 |
| 0.79166666666666696 | 148.5 |
| 0.80208333333333304 | 144.0 |
| 0.8125 | 139.0 |
| 0.82291666666666696 | 132.5 |
| 0.83333333333333304 | 126.0 |
| 0.84375 | 123.0 |
| 0.85416666666666696 | 117.5 |
| 0.86458333333333304 | 108.5 |
| 0.875 | 106.5 |
| 0.88541666666666696 | 105.5 |
| 0.89583333333333304 | 103.0 |
| 0.90625 | 99.0 |
| 0.91666666666666696 | 95.5 |
| 0.92708333333333304 | 99.0 |
| 0.9375 | 101.5 |
| 0.94791666666666696 | 98.0 |
| 0.95833333333333304 | 90.5 |
| 0.96875 | 90.0 |
| 0.97916666666666696 | 89.0 |
| 0.98958333333333304 | 84.5 |
### Chart
| Category | |
|---|---|
| 0 | 81.5 |
| 1.0416666666666666E-2 | 83.5 |
| 2.0833333333333301E-2 | 81.0 |
| 3.125E-2 | 83.5 |
| 4.1666666666666699E-2 | 88.0 |
| 5.2083333333333301E-2 | 88.0 |
| 6.25E-2 | 85.5 |
| 7.2916666666666699E-2 | 83.0 |
| 8.3333333333333301E-2 | 82.5 |
| 9.375E-2 | 83.0 |
| 0.104166666666667 | 83.5 |
| 0.114583333333333 | 78.5 |
| 0.125 | 70.5 |
| 0.13541666666666699 | 73.5 |
| 0.14583333333333301 | 86.0 |
| 0.15625 | 89.0 |
| 0.16666666666666699 | 90.0 |
| 0.17708333333333301 | 91.5 |
| 0.1875 | 91.0 |
| 0.19791666666666699 | 92.5 |
| 0.20833333333333301 | 93.5 |
| 0.21875 | 96.5 |
| 0.22916666666666699 | 98.5 |
| 0.23958333333333301 | 97.5 |
| 0.25 | 98.5 |
| 0.26041666666666702 | 96.5 |
| 0.27083333333333298 | 93.0 |
| 0.28125 | 94.5 |
| 0.29166666666666702 | 96.5 |
| 0.30208333333333298 | 95.0 |
| 0.3125 | 92.5 |
| 0.32291666666666702 | 92.5 |
| 0.33333333333333298 | 91.5 |
| 0.34375 | 100.0 |
| 0.35416666666666702 | 130.5 |
| 0.36458333333333298 | 154.5 |
| 0.375 | 156.5 |
| 0.38541666666666702 | 160.0 |
| 0.39583333333333298 | 168.0 |
| 0.40625 | 173.5 |
| 0.41666666666666702 | 169.0 |
| 0.42708333333333298 | 160.5 |
| 0.4375 | 159.5 |
| 0.44791666666666702 | 158.0 |
| 0.45833333333333298 | 152.0 |
| 0.46875 | 148.0 |
| 0.47916666666666702 | 144.0 |
| 0.48958333333333298 | 136.0 |
| 0.5 | 126.0 |
| 0.51041666666666696 | 119.0 |
| 0.52083333333333304 | 121.0 |
| 0.53125 | 129.0 |
| 0.54166666666666696 | 131.0 |
| 0.55208333333333304 | 132.0 |
| 0.5625 | 134.5 |
| 0.57291666666666696 | 131.5 |
| 0.58333333333333304 | 128.0 |
| 0.59375 | 129.5 |
| 0.60416666666666696 | 146.0 |
| 0.61458333333333304 | 155.5 |
| 0.625 | 163.5 |
| 0.63541666666666696 | 160.5 |
| 0.64583333333333304 | 147.5 |
| 0.65625 | 129.5 |
| 0.66666666666666696 | 117.5 |
| 0.67708333333333304 | 112.5 |
| 0.6875 | 103.5 |
| 0.69791666666666696 | 101.5 |
| 0.70833333333333304 | 103.0 |
| 0.71875 | 99.5 |
| 0.72916666666666696 | 92.0 |
| 0.73958333333333304 | 85.5 |
| 0.75 | 86.5 |
| 0.76041666666666696 | 91.5 |
| 0.77083333333333304 | 102.5 |
| 0.78125 | 125.5 |
| 0.79166666666666696 | 144.0 |
| 0.80208333333333304 | 148.0 |
| 0.8125 | 140.5 |
| 0.82291666666666696 | 132.5 |
| 0.83333333333333304 | 135.5 |
| 0.84375 | 142.0 |
| 0.85416666666666696 | 145.0 |
| 0.86458333333333304 | 141.5 |
| 0.875 | 136.0 |
| 0.88541666666666696 | 136.5 |
| 0.89583333333333304 | 142.5 |
| 0.90625 | 142.0 |
| 0.91666666666666696 | 135.5 |
| 0.92708333333333304 | 123.0 |
| 0.9375 | 103.0 |
| 0.94791666666666696 | 93.0 |
| 0.95833333333333304 | 98.0 |
| 0.96875 | 95.5 |
| 0.97916666666666696 | 88.0 |
| 0.98958333333333304 | 82.0 |
### Chart
| Category | |
|---|---|
| 0 | 80.0 |
| 1.0416666666666666E-2 | 77.5 |
| 2.0833333333333301E-2 | 74.0 |
| 3.125E-2 | 75.0 |
| 4.1666666666666699E-2 | 77.0 |
| 5.2083333333333301E-2 | 76.0 |
| 6.25E-2 | 77.0 |
| 7.2916666666666699E-2 | 80.0 |
| 8.3333333333333301E-2 | 79.5 |
| 9.375E-2 | 82.0 |
| 0.104166666666667 | 85.5 |
| 0.114583333333333 | 84.5 |
| 0.125 | 85.0 |
| 0.13541666666666699 | 88.0 |
| 0.14583333333333301 | 88.5 |
| 0.15625 | 87.5 |
| 0.16666666666666699 | 86.5 |
| 0.17708333333333301 | 87.5 |
| 0.1875 | 83.5 |
| 0.19791666666666699 | 79.5 |
| 0.20833333333333301 | 80.5 |
| 0.21875 | 81.5 |
| 0.22916666666666699 | 81.5 |
| 0.23958333333333301 | 83.0 |
| 0.25 | 86.0 |
| 0.26041666666666702 | 89.5 |
| 0.27083333333333298 | 90.5 |
| 0.28125 | 89.0 |
| 0.29166666666666702 | 87.0 |
| 0.30208333333333298 | 83.5 |
| 0.3125 | 82.5 |
| 0.32291666666666702 | 81.5 |
| 0.33333333333333298 | 83.5 |
| 0.34375 | 96.0 |
| 0.35416666666666702 | 124.0 |
| 0.36458333333333298 | 148.5 |
| 0.375 | 161.5 |
| 0.38541666666666702 | 160.0 |
| 0.39583333333333298 | 144.0 |
| 0.40625 | 136.0 |
| 0.41666666666666702 | 138.5 |
| 0.42708333333333298 | 135.0 |
| 0.4375 | 133.0 |
| 0.44791666666666702 | 130.5 |
| 0.45833333333333298 | 127.5 |
| 0.46875 | 125.5 |
| 0.47916666666666702 | 118.0 |
| 0.48958333333333298 | 104.5 |
| 0.5 | 95.5 |
| 0.51041666666666696 | 94.5 |
| 0.52083333333333304 | 102.0 |
| 0.53125 | 118.0 |
| 0.54166666666666696 | 124.0 |
| 0.55208333333333304 | 113.0 |
| 0.5625 | 101.0 |
| 0.57291666666666696 | 100.5 |
| 0.58333333333333304 | 110.5 |
| 0.59375 | 125.5 |
| 0.60416666666666696 | 128.5 |
| 0.61458333333333304 | 120.0 |
| 0.625 | 111.0 |
| 0.63541666666666696 | 104.5 |
| 0.64583333333333304 | 102.5 |
| 0.65625 | 104.0 |
| 0.66666666666666696 | 107.5 |
| 0.67708333333333304 | 111.0 |
| 0.6875 | 112.0 |
| 0.69791666666666696 | 111.5 |
| 0.70833333333333304 | 108.0 |
| 0.71875 | 105.0 |
| 0.72916666666666696 | 103.0 |
| 0.73958333333333304 | 97.0 |
| 0.75 | 93.5 |
| 0.76041666666666696 | 96.0 |
| 0.77083333333333304 | 106.5 |
| 0.78125 | 119.0 |
| 0.79166666666666696 | 126.0 |
| 0.80208333333333304 | 133.5 |
| 0.8125 | 142.5 |
| 0.82291666666666696 | 150.0 |
| 0.83333333333333304 | 148.5 |
| 0.84375 | 142.0 |
| 0.85416666666666696 | 135.5 |
| 0.86458333333333304 | 133.0 |
| 0.875 | 132.0 |
| 0.88541666666666696 | 131.0 |
| 0.89583333333333304 | 125.0 |
| 0.90625 | 117.0 |
| 0.91666666666666696 | 112.5 |
| 0.92708333333333304 | 108.5 |
| 0.9375 | 101.5 |
| 0.94791666666666696 | 95.5 |
| 0.95833333333333304 | 87.0 |
| 0.96875 | 81.5 |
| 0.97916666666666696 | 80.5 |
| 0.98958333333333304 | 79.5 |
### Chart
| Category | |
|---|---|
| 0 | 119.0 |
| 1.0416666666666666E-2 | 121.0 |
| 2.0833333333333301E-2 | 117.0 |
| 3.125E-2 | 116.5 |
| 4.1666666666666699E-2 | 114.0 |
| 5.2083333333333301E-2 | 105.5 |
| 6.25E-2 | 101.5 |
| 7.2916666666666699E-2 | 105.0 |
| 8.3333333333333301E-2 | 102.5 |
| 9.375E-2 | 98.0 |
| 0.104166666666667 | 95.5 |
| 0.114583333333333 | 95.5 |
| 0.125 | 96.0 |
| 0.13541666666666699 | 94.5 |
| 0.14583333333333301 | 93.5 |
| 0.15625 | 93.5 |
| 0.16666666666666699 | 92.0 |
| 0.17708333333333301 | 88.0 |
| 0.1875 | 84.5 |
| 0.19791666666666699 | 84.5 |
| 0.20833333333333301 | 83.5 |
| 0.21875 | 83.0 |
| 0.22916666666666699 | 83.5 |
| 0.23958333333333301 | 85.0 |
| 0.25 | 88.5 |
| 0.26041666666666702 | 91.5 |
| 0.27083333333333298 | 94.0 |
| 0.28125 | 91.0 |
| 0.29166666666666702 | 84.5 |
| 0.30208333333333298 | 80.0 |
| 0.3125 | 78.0 |
| 0.32291666666666702 | 81.0 |
| 0.33333333333333298 | 85.5 |
| 0.34375 | 89.5 |
| 0.35416666666666702 | 93.5 |
| 0.36458333333333298 | 97.0 |
| 0.375 | 97.5 |
| 0.38541666666666702 | 99.0 |
| 0.39583333333333298 | 106.0 |
| 0.40625 | 117.5 |
| 0.41666666666666702 | 123.0 |
| 0.42708333333333298 | 124.5 |
| 0.4375 | 130.0 |
| 0.44791666666666702 | 130.5 |
| 0.45833333333333298 | 128.0 |
| 0.46875 | 125.0 |
| 0.47916666666666702 | 120.0 |
| 0.48958333333333298 | 118.0 |
| 0.5 | 123.5 |
| 0.51041666666666696 | 124.0 |
| 0.52083333333333304 | 118.5 |
| 0.53125 | 112.5 |
| 0.54166666666666696 | 112.5 |
| 0.55208333333333304 | 122.0 |
| 0.5625 | 134.5 |
| 0.57291666666666696 | 137.0 |
| 0.58333333333333304 | 130.5 |
| 0.59375 | 130.0 |
| 0.60416666666666696 | 140.0 |
| 0.61458333333333304 | 148.5 |
| 0.625 | 153.5 |
| 0.63541666666666696 | 153.5 |
| 0.64583333333333304 | 145.5 |
| 0.65625 | 138.0 |
| 0.66666666666666696 | 131.0 |
| 0.67708333333333304 | 123.5 |
| 0.6875 | 122.0 |
| 0.69791666666666696 | 118.5 |
| 0.70833333333333304 | 111.0 |
| 0.71875 | 105.5 |
| 0.72916666666666696 | 109.0 |
| 0.73958333333333304 | 111.5 |
| 0.75 | 103.5 |
| 0.76041666666666696 | 109.5 |
| 0.77083333333333304 | 117.5 |
| 0.78125 | 132.0 |
| 0.79166666666666696 | 154.5 |
| 0.80208333333333304 | 163.5 |
| 0.8125 | 135.0 |
| 0.82291666666666696 | 144.0 |
| 0.83333333333333304 | 144.5 |
| 0.84375 | 146.5 |
| 0.85416666666666696 | 147.5 |
| 0.86458333333333304 | 144.0 |
| 0.875 | 139.5 |
| 0.88541666666666696 | 142.5 |
| 0.89583333333333304 | 145.5 |
| 0.90625 | 142.0 |
| 0.91666666666666696 | 131.0 |
| 0.92708333333333304 | 117.5 |
| 0.9375 | 112.5 |
| 0.94791666666666696 | 118.5 |
| 0.95833333333333304 | 122.5 |
| 0.96875 | 120.5 |
| 0.97916666666666696 | 116.5 |
| 0.98958333333333304 | 114.0 |
### Chart
| Category | |
|---|---|
| 0 | 88.5 |
| 1.0416666666666666E-2 | 85.5 |
| 2.0833333333333301E-2 | 83.5 |
| 3.125E-2 | 80.0 |
| 4.1666666666666699E-2 | 77.5 |
| 5.2083333333333301E-2 | 76.0 |
| 6.25E-2 | 75.0 |
| 7.2916666666666699E-2 | 75.5 |
| 8.3333333333333301E-2 | 78.5 |
| 9.375E-2 | 77.5 |
| 0.104166666666667 | 78.0 |
| 0.114583333333333 | 81.0 |
| 0.125 | 82.0 |
| 0.13541666666666699 | 83.0 |
| 0.14583333333333301 | 85.5 |
| 0.15625 | 86.5 |
| 0.16666666666666699 | 85.0 |
| 0.17708333333333301 | 85.5 |
| 0.1875 | 85.5 |
| 0.19791666666666699 | 83.5 |
| 0.20833333333333301 | 82.0 |
| 0.21875 | 79.0 |
| 0.22916666666666699 | 74.5 |
| 0.23958333333333301 | 73.5 |
| 0.25 | 75.0 |
| 0.26041666666666702 | 74.5 |
| 0.27083333333333298 | 77.5 |
| 0.28125 | 80.0 |
| 0.29166666666666702 | 75.5 |
| 0.30208333333333298 | 76.5 |
| 0.3125 | 80.5 |
| 0.32291666666666702 | 79.0 |
| 0.33333333333333298 | 83.0 |
| 0.34375 | 105.5 |
| 0.35416666666666702 | 126.0 |
| 0.36458333333333298 | 140.5 |
| 0.375 | 154.0 |
| 0.38541666666666702 | 153.0 |
| 0.39583333333333298 | 138.5 |
| 0.40625 | 123.0 |
| 0.41666666666666702 | 111.0 |
| 0.42708333333333298 | 106.0 |
| 0.4375 | 102.5 |
| 0.44791666666666702 | 96.5 |
| 0.45833333333333298 | 94.5 |
| 0.46875 | 99.0 |
| 0.47916666666666702 | 100.0 |
| 0.48958333333333298 | 96.0 |
| 0.5 | 94.0 |
| 0.51041666666666696 | 96.5 |
| 0.52083333333333304 | 103.5 |
| 0.53125 | 117.0 |
| 0.54166666666666696 | 126.5 |
| 0.55208333333333304 | 121.5 |
| 0.5625 | 110.0 |
| 0.57291666666666696 | 101.0 |
| 0.58333333333333304 | 93.5 |
| 0.59375 | 91.5 |
| 0.60416666666666696 | 92.5 |
| 0.61458333333333304 | 94.0 |
| 0.625 | 93.0 |
| 0.63541666666666696 | 94.5 |
| 0.64583333333333304 | 101.0 |
| 0.65625 | 106.0 |
| 0.66666666666666696 | 106.5 |
| 0.67708333333333304 | 107.5 |
| 0.6875 | 114.5 |
| 0.69791666666666696 | 120.5 |
| 0.70833333333333304 | 122.5 |
| 0.71875 | 121.0 |
| 0.72916666666666696 | 116.5 |
| 0.73958333333333304 | 113.0 |
| 0.75 | 107.0 |
| 0.76041666666666696 | 107.5 |
| 0.77083333333333304 | 106.5 |
| 0.78125 | 108.5 |
| 0.79166666666666696 | 113.0 |
| 0.80208333333333304 | 115.5 |
| 0.8125 | 121.5 |
| 0.82291666666666696 | 132.0 |
| 0.83333333333333304 | 137.5 |
| 0.84375 | 142.0 |
| 0.85416666666666696 | 137.5 |
| 0.86458333333333304 | 132.0 |
| 0.875 | 124.0 |
| 0.88541666666666696 | 117.5 |
| 0.89583333333333304 | 118.5 |
| 0.90625 | 120.5 |
| 0.91666666666666696 | 116.5 |
| 0.92708333333333304 | 110.5 |
| 0.9375 | 106.0 |
| 0.94791666666666696 | 101.0 |
| 0.95833333333333304 | 94.5 |
| 0.96875 | 90.0 |
| 0.97916666666666696 | 90.5 |
| 0.98958333333333304 | 91.5 |Case 5
Case 4
Case 6
(mg/dL)
(mg/dL)
(mg/dL)
(mg/dL)
(mg/dL)
(mg/dL)
### Chart
| Category | |
|---|---|
| 0 | 73.0 |
| 1.0416666666666666E-2 | 77.5 |
| 2.0833333333333301E-2 | 78.5 |
| 3.125E-2 | 76.0 |
| 4.1666666666666699E-2 | 75.0 |
| 5.2083333333333301E-2 | 73.0 |
| 6.25E-2 | 67.5 |
| 7.2916666666666699E-2 | 59.5 |
| 8.3333333333333301E-2 | 60.0 |
| 9.375E-2 | 68.5 |
| 0.104166666666667 | 70.5 |
| 0.114583333333333 | 68.0 |
| 0.125 | 69.0 |
| 0.13541666666666699 | 68.0 |
| 0.14583333333333301 | 65.0 |
| 0.15625 | 62.5 |
| 0.16666666666666699 | 62.5 |
| 0.17708333333333301 | 62.5 |
| 0.1875 | 63.5 |
| 0.19791666666666699 | 61.5 |
| 0.20833333333333301 | 61.0 |
| 0.21875 | 60.0 |
| 0.22916666666666699 | 59.5 |
| 0.23958333333333301 | 60.0 |
| 0.25 | 61.0 |
| 0.26041666666666702 | 59.0 |
| 0.27083333333333298 | 56.0 |
| 0.28125 | 54.5 |
| 0.29166666666666702 | 53.5 |
| 0.30208333333333298 | 54.0 |
| 0.3125 | 55.5 |
| 0.32291666666666702 | 53.5 |
| 0.33333333333333298 | 54.5 |
| 0.34375 | 64.5 |
| 0.35416666666666702 | 78.5 |
| 0.36458333333333298 | 89.0 |
| 0.375 | 93.0 |
| 0.38541666666666702 | 94.5 |
| 0.39583333333333298 | 97.0 |
| 0.40625 | 94.5 |
| 0.41666666666666702 | 101.0 |
| 0.42708333333333298 | 98.5 |
| 0.4375 | 95.5 |
| 0.44791666666666702 | 88.0 |
| 0.45833333333333298 | 80.5 |
| 0.46875 | 75.5 |
| 0.47916666666666702 | 68.5 |
| 0.48958333333333298 | 62.0 |
| 0.5 | 55.5 |
| 0.51041666666666696 | 56.0 |
| 0.52083333333333304 | 57.5 |
| 0.53125 | 63.5 |
| 0.54166666666666696 | 69.5 |
| 0.55208333333333304 | 70.0 |
| 0.5625 | 69.0 |
| 0.57291666666666696 | 73.5 |
| 0.58333333333333304 | 78.5 |
| 0.59375 | 80.5 |
| 0.60416666666666696 | 79.5 |
| 0.61458333333333304 | 77.5 |
| 0.625 | 74.5 |
| 0.63541666666666696 | 71.5 |
| 0.64583333333333304 | 70.5 |
| 0.65625 | 64.0 |
| 0.66666666666666696 | 63.5 |
| 0.67708333333333304 | 65.5 |
| 0.6875 | 66.5 |
| 0.69791666666666696 | 65.5 |
| 0.70833333333333304 | 64.5 |
| 0.71875 | 64.5 |
| 0.72916666666666696 | 65.0 |
| 0.73958333333333304 | 61.5 |
| 0.75 | 63.5 |
| 0.76041666666666696 | 67.0 |
| 0.77083333333333304 | 72.5 |
| 0.78125 | 90.0 |
| 0.79166666666666696 | 95.0 |
| 0.80208333333333304 | 98.0 |
| 0.8125 | 104.0 |
| 0.82291666666666696 | 108.5 |
| 0.83333333333333304 | 106.5 |
| 0.84375 | 98.0 |
| 0.85416666666666696 | 92.5 |
| 0.86458333333333304 | 90.5 |
| 0.875 | 91.5 |
| 0.88541666666666696 | 90.0 |
| 0.89583333333333304 | 90.0 |
| 0.90625 | 92.5 |
| 0.91666666666666696 | 87.5 |
| 0.92708333333333304 | 79.0 |
| 0.9375 | 73.5 |
| 0.94791666666666696 | 72.0 |
| 0.95833333333333304 | 75.0 |
| 0.96875 | 78.0 |
| 0.97916666666666696 | 76.5 |
| 0.98958333333333304 | 73.0 |
### Chart
| Category | |
|---|---|
| 0 | 61.5 |
| 1.0416666666666666E-2 | 62.5 |
| 2.0833333333333301E-2 | 65.0 |
| 3.125E-2 | 65.0 |
| 4.1666666666666699E-2 | 65.0 |
| 5.2083333333333301E-2 | 67.0 |
| 6.25E-2 | 66.5 |
| 7.2916666666666699E-2 | 66.0 |
| 8.3333333333333301E-2 | 66.0 |
| 9.375E-2 | 62.5 |
| 0.104166666666667 | 61.5 |
| 0.114583333333333 | 65.5 |
| 0.125 | 66.0 |
| 0.13541666666666699 | 65.5 |
| 0.14583333333333301 | 66.0 |
| 0.15625 | 65.0 |
| 0.16666666666666699 | 64.5 |
| 0.17708333333333301 | 63.0 |
| 0.1875 | 60.5 |
| 0.19791666666666699 | 59.5 |
| 0.20833333333333301 | 63.0 |
| 0.21875 | 63.0 |
| 0.22916666666666699 | 62.0 |
| 0.23958333333333301 | 63.5 |
| 0.25 | 63.0 |
| 0.26041666666666702 | 61.5 |
| 0.27083333333333298 | 62.5 |
| 0.28125 | 61.0 |
| 0.29166666666666702 | 59.0 |
| 0.30208333333333298 | 60.5 |
| 0.3125 | 60.5 |
| 0.32291666666666702 | 60.5 |
| 0.33333333333333298 | 64.5 |
| 0.34375 | 80.5 |
| 0.35416666666666702 | 100.0 |
| 0.36458333333333298 | 112.0 |
| 0.375 | 115.0 |
| 0.38541666666666702 | 110.0 |
| 0.39583333333333298 | 100.5 |
| 0.40625 | 93.5 |
| 0.41666666666666702 | 99.5 |
| 0.42708333333333298 | 89.0 |
| 0.4375 | 78.0 |
| 0.44791666666666702 | 71.5 |
| 0.45833333333333298 | 66.0 |
| 0.46875 | 62.0 |
| 0.47916666666666702 | 58.0 |
| 0.48958333333333298 | 56.0 |
| 0.5 | 55.5 |
| 0.51041666666666696 | 62.5 |
| 0.52083333333333304 | 74.5 |
| 0.53125 | 82.0 |
| 0.54166666666666696 | 86.5 |
| 0.55208333333333304 | 89.0 |
| 0.5625 | 86.0 |
| 0.57291666666666696 | 79.5 |
| 0.58333333333333304 | 75.0 |
| 0.59375 | 73.5 |
| 0.60416666666666696 | 72.5 |
| 0.61458333333333304 | 70.0 |
| 0.625 | 68.0 |
| 0.63541666666666696 | 67.5 |
| 0.64583333333333304 | 67.0 |
| 0.65625 | 66.5 |
| 0.66666666666666696 | 64.5 |
| 0.67708333333333304 | 62.0 |
| 0.6875 | 61.0 |
| 0.69791666666666696 | 60.5 |
| 0.70833333333333304 | 60.5 |
| 0.71875 | 60.0 |
| 0.72916666666666696 | 60.0 |
| 0.73958333333333304 | 59.5 |
| 0.75 | 62.0 |
| 0.76041666666666696 | 81.0 |
| 0.77083333333333304 | 104.0 |
| 0.78125 | 117.5 |
| 0.79166666666666696 | 124.5 |
| 0.80208333333333304 | 123.5 |
| 0.8125 | 114.0 |
| 0.82291666666666696 | 104.5 |
| 0.83333333333333304 | 96.5 |
| 0.84375 | 89.0 |
| 0.85416666666666696 | 83.0 |
| 0.86458333333333304 | 74.5 |
| 0.875 | 67.5 |
| 0.88541666666666696 | 67.0 |
| 0.89583333333333304 | 68.0 |
| 0.90625 | 64.0 |
| 0.91666666666666696 | 60.0 |
| 0.92708333333333304 | 55.5 |
| 0.9375 | 59.0 |
| 0.94791666666666696 | 62.0 |
| 0.95833333333333304 | 61.0 |
| 0.96875 | 63.0 |
| 0.97916666666666696 | 64.5 |
| 0.98958333333333304 | 63.5 |
### Chart
| Category | |
|---|---|
| 0 | 90.5 |
| 1.0416666666666666E-2 | 92.5 |
| 2.0833333333333301E-2 | 87.5 |
| 3.125E-2 | 85.5 |
| 4.1666666666666699E-2 | 86.5 |
| 5.2083333333333301E-2 | 85.5 |
| 6.25E-2 | 84.0 |
| 7.2916666666666699E-2 | 81.0 |
| 8.3333333333333301E-2 | 77.5 |
| 9.375E-2 | 78.5 |
| 0.104166666666667 | 83.5 |
| 0.114583333333333 | 87.0 |
| 0.125 | 83.5 |
| 0.13541666666666699 | 77.5 |
| 0.14583333333333301 | 80.0 |
| 0.15625 | 85.5 |
| 0.16666666666666699 | 86.5 |
| 0.17708333333333301 | 87.0 |
| 0.1875 | 84.0 |
| 0.19791666666666699 | 83.0 |
| 0.20833333333333301 | 85.5 |
| 0.21875 | 86.5 |
| 0.22916666666666699 | 86.5 |
| 0.23958333333333301 | 85.0 |
| 0.25 | 82.0 |
| 0.26041666666666702 | 83.0 |
| 0.27083333333333298 | 90.0 |
| 0.28125 | 96.0 |
| 0.29166666666666702 | 97.5 |
| 0.30208333333333298 | 97.0 |
| 0.3125 | 95.5 |
| 0.32291666666666702 | 96.0 |
| 0.33333333333333298 | 101.5 |
| 0.34375 | 120.5 |
| 0.35416666666666702 | 157.0 |
| 0.36458333333333298 | 180.5 |
| 0.375 | 185.5 |
| 0.38541666666666702 | 183.5 |
| 0.39583333333333298 | 178.5 |
| 0.40625 | 172.0 |
| 0.41666666666666702 | 161.5 |
| 0.42708333333333298 | 150.0 |
| 0.4375 | 141.0 |
| 0.44791666666666702 | 135.5 |
| 0.45833333333333298 | 132.0 |
| 0.46875 | 133.0 |
| 0.47916666666666702 | 127.5 |
| 0.48958333333333298 | 116.5 |
| 0.5 | 107.5 |
| 0.51041666666666696 | 103.0 |
| 0.52083333333333304 | 106.5 |
| 0.53125 | 113.5 |
| 0.54166666666666696 | 117.0 |
| 0.55208333333333304 | 117.0 |
| 0.5625 | 114.5 |
| 0.57291666666666696 | 114.5 |
| 0.58333333333333304 | 116.5 |
| 0.59375 | 106.5 |
| 0.60416666666666696 | 124.5 |
| 0.61458333333333304 | 127.5 |
| 0.625 | 135.5 |
| 0.63541666666666696 | 137.0 |
| 0.64583333333333304 | 134.5 |
| 0.65625 | 128.0 |
| 0.66666666666666696 | 120.0 |
| 0.67708333333333304 | 119.5 |
| 0.6875 | 123.0 |
| 0.69791666666666696 | 125.0 |
| 0.70833333333333304 | 116.5 |
| 0.71875 | 99.5 |
| 0.72916666666666696 | 95.5 |
| 0.73958333333333304 | 101.0 |
| 0.75 | 102.0 |
| 0.76041666666666696 | 107.0 |
| 0.77083333333333304 | 133.5 |
| 0.78125 | 175.5 |
| 0.79166666666666696 | 200.5 |
| 0.80208333333333304 | 206.0 |
| 0.8125 | 201.0 |
| 0.82291666666666696 | 178.0 |
| 0.83333333333333304 | 159.5 |
| 0.84375 | 160.5 |
| 0.85416666666666696 | 150.5 |
| 0.86458333333333304 | 129.0 |
| 0.875 | 115.5 |
| 0.88541666666666696 | 114.0 |
| 0.89583333333333304 | 112.5 |
| 0.90625 | 105.0 |
| 0.91666666666666696 | 98.0 |
| 0.92708333333333304 | 97.5 |
| 0.9375 | 95.0 |
| 0.94791666666666696 | 84.5 |
| 0.95833333333333304 | 80.5 |
| 0.96875 | 84.0 |
| 0.97916666666666696 | 84.0 |
| 0.98958333333333304 | 86.0 |
### Chart
| Category | |
|---|---|
| 0 | 84.5 |
| 1.0416666666666666E-2 | 82.5 |
| 2.0833333333333301E-2 | 83.0 |
| 3.125E-2 | 83.5 |
| 4.1666666666666699E-2 | 82.5 |
| 5.2083333333333301E-2 | 80.5 |
| 6.25E-2 | 80.0 |
| 7.2916666666666699E-2 | 80.0 |
| 8.3333333333333301E-2 | 81.5 |
| 9.375E-2 | 82.0 |
| 0.104166666666667 | 78.5 |
| 0.114583333333333 | 77.0 |
| 0.125 | 80.0 |
| 0.13541666666666699 | 82.0 |
| 0.14583333333333301 | 79.5 |
| 0.15625 | 78.5 |
| 0.16666666666666699 | 78.0 |
| 0.17708333333333301 | 77.5 |
| 0.1875 | 80.0 |
| 0.19791666666666699 | 80.5 |
| 0.20833333333333301 | 79.0 |
| 0.21875 | 75.0 |
| 0.22916666666666699 | 70.5 |
| 0.23958333333333301 | 72.5 |
| 0.25 | 78.5 |
| 0.26041666666666702 | 78.0 |
| 0.27083333333333298 | 80.5 |
| 0.28125 | 85.0 |
| 0.29166666666666702 | 85.5 |
| 0.30208333333333298 | 83.0 |
| 0.3125 | 86.0 |
| 0.32291666666666702 | 91.5 |
| 0.33333333333333298 | 93.5 |
| 0.34375 | 97.0 |
| 0.35416666666666702 | 113.5 |
| 0.36458333333333298 | 143.0 |
| 0.375 | 161.5 |
| 0.38541666666666702 | 168.0 |
| 0.39583333333333298 | 162.0 |
| 0.40625 | 148.5 |
| 0.41666666666666702 | 135.0 |
| 0.42708333333333298 | 122.5 |
| 0.4375 | 111.5 |
| 0.44791666666666702 | 104.0 |
| 0.45833333333333298 | 101.0 |
| 0.46875 | 102.0 |
| 0.47916666666666702 | 102.5 |
| 0.48958333333333298 | 99.5 |
| 0.5 | 99.5 |
| 0.51041666666666696 | 112.5 |
| 0.52083333333333304 | 133.0 |
| 0.53125 | 139.0 |
| 0.54166666666666696 | 132.0 |
| 0.55208333333333304 | 125.0 |
| 0.5625 | 117.0 |
| 0.57291666666666696 | 103.5 |
| 0.58333333333333304 | 97.0 |
| 0.59375 | 108.5 |
| 0.60416666666666696 | 115.0 |
| 0.61458333333333304 | 118.0 |
| 0.625 | 111.5 |
| 0.63541666666666696 | 111.0 |
| 0.64583333333333304 | 112.0 |
| 0.65625 | 111.5 |
| 0.66666666666666696 | 110.5 |
| 0.67708333333333304 | 107.0 |
| 0.6875 | 104.5 |
| 0.69791666666666696 | 103.0 |
| 0.70833333333333304 | 102.5 |
| 0.71875 | 101.0 |
| 0.72916666666666696 | 97.5 |
| 0.73958333333333304 | 93.5 |
| 0.75 | 93.5 |
| 0.76041666666666696 | 107.0 |
| 0.77083333333333304 | 133.0 |
| 0.78125 | 149.0 |
| 0.79166666666666696 | 142.0 |
| 0.80208333333333304 | 126.5 |
| 0.8125 | 116.0 |
| 0.82291666666666696 | 110.0 |
| 0.83333333333333304 | 107.5 |
| 0.84375 | 106.5 |
| 0.85416666666666696 | 108.0 |
| 0.86458333333333304 | 110.5 |
| 0.875 | 108.5 |
| 0.88541666666666696 | 104.0 |
| 0.89583333333333304 | 101.5 |
| 0.90625 | 95.5 |
| 0.91666666666666696 | 90.0 |
| 0.92708333333333304 | 90.5 |
| 0.9375 | 91.0 |
| 0.94791666666666696 | 90.0 |
| 0.95833333333333304 | 87.0 |
| 0.96875 | 83.0 |
| 0.97916666666666696 | 84.5 |
| 0.98958333333333304 | 85.5 |
### Chart
| Category | |
|---|---|
| 0 | 102.5 |
| 1.0416666666666666E-2 | 103.0 |
| 2.0833333333333301E-2 | 103.0 |
| 3.125E-2 | 99.0 |
| 4.1666666666666699E-2 | 96.0 |
| 5.2083333333333301E-2 | 95.0 |
| 6.25E-2 | 94.0 |
| 7.2916666666666699E-2 | 91.0 |
| 8.3333333333333301E-2 | 87.0 |
| 9.375E-2 | 82.5 |
| 0.104166666666667 | 80.0 |
| 0.114583333333333 | 78.0 |
| 0.125 | 75.5 |
| 0.13541666666666699 | 74.5 |
| 0.14583333333333301 | 74.5 |
| 0.15625 | 77.0 |
| 0.16666666666666699 | 80.0 |
| 0.17708333333333301 | 80.0 |
| 0.1875 | 80.0 |
| 0.19791666666666699 | 80.0 |
| 0.20833333333333301 | 79.5 |
| 0.21875 | 78.5 |
| 0.22916666666666699 | 79.5 |
| 0.23958333333333301 | 82.5 |
| 0.25 | 83.5 |
| 0.26041666666666702 | 82.5 |
| 0.27083333333333298 | 81.5 |
| 0.28125 | 82.0 |
| 0.29166666666666702 | 85.0 |
| 0.30208333333333298 | 88.5 |
| 0.3125 | 88.0 |
| 0.32291666666666702 | 86.0 |
| 0.33333333333333298 | 92.0 |
| 0.34375 | 113.0 |
| 0.35416666666666702 | 135.0 |
| 0.36458333333333298 | 137.5 |
| 0.375 | 129.0 |
| 0.38541666666666702 | 123.5 |
| 0.39583333333333298 | 124.0 |
| 0.40625 | 124.5 |
| 0.41666666666666702 | 127.0 |
| 0.42708333333333298 | 127.5 |
| 0.4375 | 122.0 |
| 0.44791666666666702 | 110.5 |
| 0.45833333333333298 | 99.0 |
| 0.46875 | 96.5 |
| 0.47916666666666702 | 104.0 |
| 0.48958333333333298 | 110.5 |
| 0.5 | 113.0 |
| 0.51041666666666696 | 118.0 |
| 0.52083333333333304 | 125.5 |
| 0.53125 | 132.0 |
| 0.54166666666666696 | 136.5 |
| 0.55208333333333304 | 137.5 |
| 0.5625 | 139.0 |
| 0.57291666666666696 | 142.0 |
| 0.58333333333333304 | 141.5 |
| 0.59375 | 136.0 |
| 0.60416666666666696 | 134.0 |
| 0.61458333333333304 | 139.0 |
| 0.625 | 140.0 |
| 0.63541666666666696 | 136.0 |
| 0.64583333333333304 | 135.0 |
| 0.65625 | 134.5 |
| 0.66666666666666696 | 132.0 |
| 0.67708333333333304 | 132.0 |
| 0.6875 | 134.0 |
| 0.69791666666666696 | 133.5 |
| 0.70833333333333304 | 129.5 |
| 0.71875 | 129.5 |
| 0.72916666666666696 | 130.5 |
| 0.73958333333333304 | 127.5 |
| 0.75 | 126.0 |
| 0.76041666666666696 | 138.0 |
| 0.77083333333333304 | 161.0 |
| 0.78125 | 172.5 |
| 0.79166666666666696 | 174.0 |
| 0.80208333333333304 | 181.5 |
| 0.8125 | 194.0 |
| 0.82291666666666696 | 199.0 |
| 0.83333333333333304 | 199.0 |
| 0.84375 | 197.5 |
| 0.85416666666666696 | 192.5 |
| 0.86458333333333304 | 189.0 |
| 0.875 | 184.0 |
| 0.88541666666666696 | 172.5 |
| 0.89583333333333304 | 165.0 |
| 0.90625 | 159.0 |
| 0.91666666666666696 | 152.0 |
| 0.92708333333333304 | 143.5 |
| 0.9375 | 134.0 |
| 0.94791666666666696 | 126.5 |
| 0.95833333333333304 | 121.5 |
| 0.96875 | 117.5 |
| 0.97916666666666696 | 112.5 |
| 0.98958333333333304 | 106.5 |
### Chart
| Category | |
|---|---|
| 0 | 81.5 |
| 1.0416666666666666E-2 | 84.0 |
| 2.0833333333333301E-2 | 87.0 |
| 3.125E-2 | 86.5 |
| 4.1666666666666699E-2 | 83.5 |
| 5.2083333333333301E-2 | 79.5 |
| 6.25E-2 | 81.0 |
| 7.2916666666666699E-2 | 84.5 |
| 8.3333333333333301E-2 | 84.5 |
| 9.375E-2 | 81.5 |
| 0.104166666666667 | 81.5 |
| 0.114583333333333 | 81.5 |
| 0.125 | 80.5 |
| 0.13541666666666699 | 80.0 |
| 0.14583333333333301 | 79.5 |
| 0.15625 | 79.0 |
| 0.16666666666666699 | 80.5 |
| 0.17708333333333301 | 82.0 |
| 0.1875 | 81.5 |
| 0.19791666666666699 | 81.5 |
| 0.20833333333333301 | 82.5 |
| 0.21875 | 81.5 |
| 0.22916666666666699 | 79.5 |
| 0.23958333333333301 | 76.0 |
| 0.25 | 77.0 |
| 0.26041666666666702 | 80.0 |
| 0.27083333333333298 | 80.5 |
| 0.28125 | 79.5 |
| 0.29166666666666702 | 78.5 |
| 0.30208333333333298 | 79.5 |
| 0.3125 | 81.0 |
| 0.32291666666666702 | 80.5 |
| 0.33333333333333298 | 81.0 |
| 0.34375 | 91.5 |
| 0.35416666666666702 | 107.5 |
| 0.36458333333333298 | 112.0 |
| 0.375 | 110.0 |
| 0.38541666666666702 | 104.0 |
| 0.39583333333333298 | 95.5 |
| 0.40625 | 93.0 |
| 0.41666666666666702 | 93.5 |
| 0.42708333333333298 | 93.0 |
| 0.4375 | 92.5 |
| 0.44791666666666702 | 88.0 |
| 0.45833333333333298 | 87.0 |
| 0.46875 | 89.0 |
| 0.47916666666666702 | 87.5 |
| 0.48958333333333298 | 88.5 |
| 0.5 | 91.5 |
| 0.51041666666666696 | 103.0 |
| 0.52083333333333304 | 112.5 |
| 0.53125 | 112.0 |
| 0.54166666666666696 | 110.5 |
| 0.55208333333333304 | 109.5 |
| 0.5625 | 113.0 |
| 0.57291666666666696 | 116.0 |
| 0.58333333333333304 | 117.0 |
| 0.59375 | 121.0 |
| 0.60416666666666696 | 122.5 |
| 0.61458333333333304 | 120.5 |
| 0.625 | 120.0 |
| 0.63541666666666696 | 121.0 |
| 0.64583333333333304 | 118.5 |
| 0.65625 | 115.5 |
| 0.66666666666666696 | 113.0 |
| 0.67708333333333304 | 108.5 |
| 0.6875 | 106.0 |
| 0.69791666666666696 | 104.0 |
| 0.70833333333333304 | 104.0 |
| 0.71875 | 104.0 |
| 0.72916666666666696 | 99.5 |
| 0.73958333333333304 | 98.0 |
| 0.75 | 96.0 |
| 0.76041666666666696 | 112.5 |
| 0.77083333333333304 | 125.5 |
| 0.78125 | 130.5 |
| 0.79166666666666696 | 134.5 |
| 0.80208333333333304 | 139.5 |
| 0.8125 | 139.5 |
| 0.82291666666666696 | 137.5 |
| 0.83333333333333304 | 135.5 |
| 0.84375 | 130.0 |
| 0.85416666666666696 | 124.0 |
| 0.86458333333333304 | 118.5 |
| 0.875 | 117.0 |
| 0.88541666666666696 | 115.0 |
| 0.89583333333333304 | 109.0 |
| 0.90625 | 100.5 |
| 0.91666666666666696 | 98.0 |
| 0.92708333333333304 | 99.5 |
| 0.9375 | 98.5 |
| 0.94791666666666696 | 92.5 |
| 0.95833333333333304 | 87.5 |
| 0.96875 | 86.5 |
| 0.97916666666666696 | 85.5 |
| 0.98958333333333304 | 82.0 |Case 7
Case 8
Case 9
(mg/dL)
(mg/dL)
(mg/dL)
(mg/dL)
(mg/dL)
(mg/dL)
### Chart
| Category | |
|---|---|
| 0 | 147.0 |
| 1.0416666666666666E-2 | 145.5 |
| 2.0833333333333301E-2 | 142.5 |
| 3.125E-2 | 137.5 |
| 4.1666666666666699E-2 | 131.0 |
| 5.2083333333333301E-2 | 126.0 |
| 6.25E-2 | 117.5 |
| 7.2916666666666699E-2 | 107.5 |
| 8.3333333333333301E-2 | 106.5 |
| 9.375E-2 | 113.5 |
| 0.104166666666667 | 112.0 |
| 0.114583333333333 | 107.0 |
| 0.125 | 107.0 |
| 0.13541666666666699 | 108.5 |
| 0.14583333333333301 | 105.0 |
| 0.15625 | 96.5 |
| 0.16666666666666699 | 98.0 |
| 0.17708333333333301 | 104.0 |
| 0.1875 | 97.5 |
| 0.19791666666666699 | 94.0 |
| 0.20833333333333301 | 93.5 |
| 0.21875 | 89.5 |
| 0.22916666666666699 | 86.0 |
| 0.23958333333333301 | 84.5 |
| 0.25 | 84.0 |
| 0.26041666666666702 | 85.5 |
| 0.27083333333333298 | 82.0 |
| 0.28125 | 76.5 |
| 0.29166666666666702 | 83.0 |
| 0.30208333333333298 | 84.5 |
| 0.3125 | 80.5 |
| 0.32291666666666702 | 81.5 |
| 0.33333333333333298 | 87.5 |
| 0.34375 | 95.0 |
| 0.35416666666666702 | 104.5 |
| 0.36458333333333298 | 124.0 |
| 0.375 | 151.0 |
| 0.38541666666666702 | 176.5 |
| 0.39583333333333298 | 194.0 |
| 0.40625 | 202.5 |
| 0.41666666666666702 | 195.5 |
| 0.42708333333333298 | 169.5 |
| 0.4375 | 155.5 |
| 0.44791666666666702 | 151.5 |
| 0.45833333333333298 | 148.5 |
| 0.46875 | 142.5 |
| 0.47916666666666702 | 135.5 |
| 0.48958333333333298 | 131.0 |
| 0.5 | 127.5 |
| 0.51041666666666696 | 121.0 |
| 0.52083333333333304 | 124.5 |
| 0.53125 | 141.5 |
| 0.54166666666666696 | 162.0 |
| 0.55208333333333304 | 182.0 |
| 0.5625 | 190.5 |
| 0.57291666666666696 | 188.0 |
| 0.58333333333333304 | 187.0 |
| 0.59375 | 188.0 |
| 0.60416666666666696 | 190.5 |
| 0.61458333333333304 | 188.0 |
| 0.625 | 174.0 |
| 0.63541666666666696 | 163.0 |
| 0.64583333333333304 | 164.0 |
| 0.65625 | 169.5 |
| 0.66666666666666696 | 175.5 |
| 0.67708333333333304 | 185.0 |
| 0.6875 | 193.0 |
| 0.69791666666666696 | 196.5 |
| 0.70833333333333304 | 180.5 |
| 0.71875 | 179.0 |
| 0.72916666666666696 | 183.0 |
| 0.73958333333333304 | 186.5 |
| 0.75 | 185.0 |
| 0.76041666666666696 | 177.5 |
| 0.77083333333333304 | 170.5 |
| 0.78125 | 178.5 |
| 0.79166666666666696 | 187.5 |
| 0.80208333333333304 | 182.0 |
| 0.8125 | 177.5 |
| 0.82291666666666696 | 170.0 |
| 0.83333333333333304 | 165.5 |
| 0.84375 | 165.0 |
| 0.85416666666666696 | 163.0 |
| 0.86458333333333304 | 174.5 |
| 0.875 | 191.0 |
| 0.88541666666666696 | 197.5 |
| 0.89583333333333304 | 194.0 |
| 0.90625 | 179.5 |
| 0.91666666666666696 | 169.5 |
| 0.92708333333333304 | 164.0 |
| 0.9375 | 152.5 |
| 0.94791666666666696 | 142.0 |
| 0.95833333333333304 | 143.0 |
| 0.96875 | 149.5 |
| 0.97916666666666696 | 150.5 |
| 0.98958333333333304 | 149.0 |
### Chart
| Category | |
|---|---|
| 0 | 111.0 |
| 1.0416666666666666E-2 | 111.0 |
| 2.0833333333333301E-2 | 113.0 |
| 3.125E-2 | 113.5 |
| 4.1666666666666699E-2 | 111.0 |
| 5.2083333333333301E-2 | 105.5 |
| 6.25E-2 | 100.0 |
| 7.2916666666666699E-2 | 97.0 |
| 8.3333333333333301E-2 | 96.0 |
| 9.375E-2 | 103.0 |
| 0.104166666666667 | 110.5 |
| 0.114583333333333 | 115.0 |
| 0.125 | 116.5 |
| 0.13541666666666699 | 116.0 |
| 0.14583333333333301 | 117.0 |
| 0.15625 | 115.5 |
| 0.16666666666666699 | 112.5 |
| 0.17708333333333301 | 112.5 |
| 0.1875 | 117.0 |
| 0.19791666666666699 | 122.5 |
| 0.20833333333333301 | 126.0 |
| 0.21875 | 125.5 |
| 0.22916666666666699 | 125.5 |
| 0.23958333333333301 | 125.0 |
| 0.25 | 125.5 |
| 0.26041666666666702 | 129.0 |
| 0.27083333333333298 | 130.0 |
| 0.28125 | 134.0 |
| 0.29166666666666702 | 138.0 |
| 0.30208333333333298 | 139.0 |
| 0.3125 | 138.5 |
| 0.32291666666666702 | 137.5 |
| 0.33333333333333298 | 135.0 |
| 0.34375 | 135.5 |
| 0.35416666666666702 | 146.5 |
| 0.36458333333333298 | 165.0 |
| 0.375 | 179.0 |
| 0.38541666666666702 | 188.0 |
| 0.39583333333333298 | 197.0 |
| 0.40625 | 203.5 |
| 0.41666666666666702 | 201.0 |
| 0.42708333333333298 | 186.0 |
| 0.4375 | 172.5 |
| 0.44791666666666702 | 162.0 |
| 0.45833333333333298 | 150.0 |
| 0.46875 | 139.0 |
| 0.47916666666666702 | 127.5 |
| 0.48958333333333298 | 118.5 |
| 0.5 | 117.0 |
| 0.51041666666666696 | 122.5 |
| 0.52083333333333304 | 132.5 |
| 0.53125 | 142.0 |
| 0.54166666666666696 | 144.0 |
| 0.55208333333333304 | 144.5 |
| 0.5625 | 149.0 |
| 0.57291666666666696 | 149.5 |
| 0.58333333333333304 | 146.5 |
| 0.59375 | 151.5 |
| 0.60416666666666696 | 153.0 |
| 0.61458333333333304 | 148.0 |
| 0.625 | 149.5 |
| 0.63541666666666696 | 152.5 |
| 0.64583333333333304 | 151.5 |
| 0.65625 | 155.0 |
| 0.66666666666666696 | 160.5 |
| 0.67708333333333304 | 161.5 |
| 0.6875 | 161.0 |
| 0.69791666666666696 | 159.5 |
| 0.70833333333333304 | 149.5 |
| 0.71875 | 146.0 |
| 0.72916666666666696 | 141.0 |
| 0.73958333333333304 | 140.5 |
| 0.75 | 145.0 |
| 0.76041666666666696 | 148.0 |
| 0.77083333333333304 | 149.0 |
| 0.78125 | 149.5 |
| 0.79166666666666696 | 153.0 |
| 0.80208333333333304 | 157.5 |
| 0.8125 | 161.0 |
| 0.82291666666666696 | 162.0 |
| 0.83333333333333304 | 160.0 |
| 0.84375 | 157.5 |
| 0.85416666666666696 | 155.0 |
| 0.86458333333333304 | 154.0 |
| 0.875 | 150.0 |
| 0.88541666666666696 | 144.5 |
| 0.89583333333333304 | 139.0 |
| 0.90625 | 138.5 |
| 0.91666666666666696 | 137.5 |
| 0.92708333333333304 | 130.5 |
| 0.9375 | 123.5 |
| 0.94791666666666696 | 121.0 |
| 0.95833333333333304 | 120.5 |
| 0.96875 | 118.5 |
| 0.97916666666666696 | 115.0 |
| 0.98958333333333304 | 112.5 |
### Chart
| Category | |
|---|---|
| 0 | 117.0 |
| 1.0416666666666666E-2 | 113.0 |
| 2.0833333333333301E-2 | 110.0 |
| 3.125E-2 | 107.0 |
| 4.1666666666666699E-2 | 103.5 |
| 5.2083333333333301E-2 | 101.0 |
| 6.25E-2 | 101.0 |
| 7.2916666666666699E-2 | 101.0 |
| 8.3333333333333301E-2 | 96.0 |
| 9.375E-2 | 90.5 |
| 0.104166666666667 | 91.0 |
| 0.114583333333333 | 94.5 |
| 0.125 | 95.5 |
| 0.13541666666666699 | 97.5 |
| 0.14583333333333301 | 102.5 |
| 0.15625 | 106.0 |
| 0.16666666666666699 | 103.0 |
| 0.17708333333333301 | 103.0 |
| 0.1875 | 107.0 |
| 0.19791666666666699 | 110.0 |
| 0.20833333333333301 | 116.5 |
| 0.21875 | 119.0 |
| 0.22916666666666699 | 114.0 |
| 0.23958333333333301 | 109.5 |
| 0.25 | 108.0 |
| 0.26041666666666702 | 106.5 |
| 0.27083333333333298 | 105.5 |
| 0.28125 | 103.0 |
| 0.29166666666666702 | 98.5 |
| 0.30208333333333298 | 101.0 |
| 0.3125 | 104.0 |
| 0.32291666666666702 | 101.0 |
| 0.33333333333333298 | 101.0 |
| 0.34375 | 109.5 |
| 0.35416666666666702 | 128.0 |
| 0.36458333333333298 | 150.5 |
| 0.375 | 161.0 |
| 0.38541666666666702 | 156.0 |
| 0.39583333333333298 | 150.5 |
| 0.40625 | 143.0 |
| 0.41666666666666702 | 133.5 |
| 0.42708333333333298 | 129.5 |
| 0.4375 | 128.0 |
| 0.44791666666666702 | 120.5 |
| 0.45833333333333298 | 110.0 |
| 0.46875 | 100.5 |
| 0.47916666666666702 | 95.5 |
| 0.48958333333333298 | 93.5 |
| 0.5 | 94.0 |
| 0.51041666666666696 | 96.0 |
| 0.52083333333333304 | 100.0 |
| 0.53125 | 105.5 |
| 0.54166666666666696 | 104.5 |
| 0.55208333333333304 | 95.5 |
| 0.5625 | 86.5 |
| 0.57291666666666696 | 89.0 |
| 0.58333333333333304 | 108.5 |
| 0.59375 | 121.5 |
| 0.60416666666666696 | 124.0 |
| 0.61458333333333304 | 119.5 |
| 0.625 | 114.5 |
| 0.63541666666666696 | 114.5 |
| 0.64583333333333304 | 117.0 |
| 0.65625 | 113.5 |
| 0.66666666666666696 | 108.0 |
| 0.67708333333333304 | 113.5 |
| 0.6875 | 124.0 |
| 0.69791666666666696 | 125.5 |
| 0.70833333333333304 | 121.5 |
| 0.71875 | 119.5 |
| 0.72916666666666696 | 117.5 |
| 0.73958333333333304 | 107.5 |
| 0.75 | 90.0 |
| 0.76041666666666696 | 80.5 |
| 0.77083333333333304 | 94.0 |
| 0.78125 | 110.0 |
| 0.79166666666666696 | 114.0 |
| 0.80208333333333304 | 117.0 |
| 0.8125 | 123.5 |
| 0.82291666666666696 | 127.0 |
| 0.83333333333333304 | 126.0 |
| 0.84375 | 131.0 |
| 0.85416666666666696 | 152.5 |
| 0.86458333333333304 | 157.0 |
| 0.875 | 158.0 |
| 0.88541666666666696 | 154.5 |
| 0.89583333333333304 | 144.0 |
| 0.90625 | 132.0 |
| 0.91666666666666696 | 131.5 |
| 0.92708333333333304 | 138.0 |
| 0.9375 | 138.5 |
| 0.94791666666666696 | 138.5 |
| 0.95833333333333304 | 137.0 |
| 0.96875 | 128.0 |
| 0.97916666666666696 | 121.0 |
| 0.98958333333333304 | 119.0 |
### Chart
| Category | |
|---|---|
| 0 | 97.0 |
| 1.0416666666666666E-2 | 93.5 |
| 2.0833333333333301E-2 | 88.5 |
| 3.125E-2 | 83.0 |
| 4.1666666666666699E-2 | 77.0 |
| 5.2083333333333301E-2 | 72.5 |
| 6.25E-2 | 71.0 |
| 7.2916666666666699E-2 | 73.0 |
| 8.3333333333333301E-2 | 76.0 |
| 9.375E-2 | 75.5 |
| 0.104166666666667 | 75.0 |
| 0.114583333333333 | 77.5 |
| 0.125 | 79.5 |
| 0.13541666666666699 | 80.5 |
| 0.14583333333333301 | 79.0 |
| 0.15625 | 78.0 |
| 0.16666666666666699 | 78.0 |
| 0.17708333333333301 | 80.5 |
| 0.1875 | 81.0 |
| 0.19791666666666699 | 80.5 |
| 0.20833333333333301 | 82.0 |
| 0.21875 | 84.0 |
| 0.22916666666666699 | 84.5 |
| 0.23958333333333301 | 86.5 |
| 0.25 | 88.0 |
| 0.26041666666666702 | 88.5 |
| 0.27083333333333298 | 90.5 |
| 0.28125 | 91.0 |
| 0.29166666666666702 | 89.0 |
| 0.30208333333333298 | 89.0 |
| 0.3125 | 88.0 |
| 0.32291666666666702 | 86.0 |
| 0.33333333333333298 | 86.0 |
| 0.34375 | 89.5 |
| 0.35416666666666702 | 103.5 |
| 0.36458333333333298 | 126.5 |
| 0.375 | 146.0 |
| 0.38541666666666702 | 155.0 |
| 0.39583333333333298 | 149.5 |
| 0.40625 | 141.0 |
| 0.41666666666666702 | 133.0 |
| 0.42708333333333298 | 129.0 |
| 0.4375 | 121.0 |
| 0.44791666666666702 | 107.5 |
| 0.45833333333333298 | 99.5 |
| 0.46875 | 96.5 |
| 0.47916666666666702 | 91.5 |
| 0.48958333333333298 | 83.5 |
| 0.5 | 80.5 |
| 0.51041666666666696 | 82.0 |
| 0.52083333333333304 | 88.0 |
| 0.53125 | 98.5 |
| 0.54166666666666696 | 105.5 |
| 0.55208333333333304 | 104.5 |
| 0.5625 | 98.0 |
| 0.57291666666666696 | 93.0 |
| 0.58333333333333304 | 94.5 |
| 0.59375 | 101.0 |
| 0.60416666666666696 | 111.5 |
| 0.61458333333333304 | 125.0 |
| 0.625 | 131.5 |
| 0.63541666666666696 | 125.5 |
| 0.64583333333333304 | 113.5 |
| 0.65625 | 104.5 |
| 0.66666666666666696 | 101.0 |
| 0.67708333333333304 | 106.5 |
| 0.6875 | 113.5 |
| 0.69791666666666696 | 115.5 |
| 0.70833333333333304 | 114.0 |
| 0.71875 | 112.0 |
| 0.72916666666666696 | 108.0 |
| 0.73958333333333304 | 104.5 |
| 0.75 | 97.5 |
| 0.76041666666666696 | 90.5 |
| 0.77083333333333304 | 95.0 |
| 0.78125 | 112.5 |
| 0.79166666666666696 | 130.5 |
| 0.80208333333333304 | 140.5 |
| 0.8125 | 142.0 |
| 0.82291666666666696 | 143.5 |
| 0.83333333333333304 | 153.0 |
| 0.84375 | 162.5 |
| 0.85416666666666696 | 151.5 |
| 0.86458333333333304 | 161.0 |
| 0.875 | 157.5 |
| 0.88541666666666696 | 146.5 |
| 0.89583333333333304 | 134.5 |
| 0.90625 | 128.0 |
| 0.91666666666666696 | 125.5 |
| 0.92708333333333304 | 126.5 |
| 0.9375 | 125.0 |
| 0.94791666666666696 | 120.0 |
| 0.95833333333333304 | 117.0 |
| 0.96875 | 112.5 |
| 0.97916666666666696 | 106.0 |
| 0.98958333333333304 | 100.0 |
### Chart
| Category | |
|---|---|
| 0 | 112.5 |
| 1.0416666666666666E-2 | 108.0 |
| 2.0833333333333301E-2 | 102.0 |
| 3.125E-2 | 93.0 |
| 4.1666666666666699E-2 | 88.0 |
| 5.2083333333333301E-2 | 88.5 |
| 6.25E-2 | 91.5 |
| 7.2916666666666699E-2 | 94.5 |
| 8.3333333333333301E-2 | 92.0 |
| 9.375E-2 | 88.5 |
| 0.104166666666667 | 87.0 |
| 0.114583333333333 | 85.5 |
| 0.125 | 86.5 |
| 0.13541666666666699 | 88.0 |
| 0.14583333333333301 | 84.0 |
| 0.15625 | 81.5 |
| 0.16666666666666699 | 79.0 |
| 0.17708333333333301 | 76.0 |
| 0.1875 | 76.0 |
| 0.19791666666666699 | 79.0 |
| 0.20833333333333301 | 80.5 |
| 0.21875 | 82.0 |
| 0.22916666666666699 | 84.5 |
| 0.23958333333333301 | 89.0 |
| 0.25 | 94.5 |
| 0.26041666666666702 | 101.0 |
| 0.27083333333333298 | 104.5 |
| 0.28125 | 102.5 |
| 0.29166666666666702 | 97.0 |
| 0.30208333333333298 | 97.0 |
| 0.3125 | 102.0 |
| 0.32291666666666702 | 106.5 |
| 0.33333333333333298 | 124.5 |
| 0.34375 | 170.5 |
| 0.35416666666666702 | 222.5 |
| 0.36458333333333298 | 246.5 |
| 0.375 | 233.0 |
| 0.38541666666666702 | 213.0 |
| 0.39583333333333298 | 194.0 |
| 0.40625 | 185.0 |
| 0.41666666666666702 | 180.5 |
| 0.42708333333333298 | 165.5 |
| 0.4375 | 150.5 |
| 0.44791666666666702 | 138.5 |
| 0.45833333333333298 | 129.0 |
| 0.46875 | 126.0 |
| 0.47916666666666702 | 121.0 |
| 0.48958333333333298 | 118.0 |
| 0.5 | 135.5 |
| 0.51041666666666696 | 176.5 |
| 0.52083333333333304 | 219.5 |
| 0.53125 | 248.5 |
| 0.54166666666666696 | 260.5 |
| 0.55208333333333304 | 257.5 |
| 0.5625 | 239.5 |
| 0.57291666666666696 | 218.0 |
| 0.58333333333333304 | 203.0 |
| 0.59375 | 196.5 |
| 0.60416666666666696 | 189.5 |
| 0.61458333333333304 | 180.0 |
| 0.625 | 170.5 |
| 0.63541666666666696 | 165.5 |
| 0.64583333333333304 | 161.0 |
| 0.65625 | 156.0 |
| 0.66666666666666696 | 146.0 |
| 0.67708333333333304 | 133.0 |
| 0.6875 | 123.5 |
| 0.69791666666666696 | 118.0 |
| 0.70833333333333304 | 119.5 |
| 0.71875 | 116.0 |
| 0.72916666666666696 | 116.0 |
| 0.73958333333333304 | 115.5 |
| 0.75 | 132.0 |
| 0.76041666666666696 | 179.5 |
| 0.77083333333333304 | 226.0 |
| 0.78125 | 258.5 |
| 0.79166666666666696 | 275.5 |
| 0.80208333333333304 | 279.5 |
| 0.8125 | 277.0 |
| 0.82291666666666696 | 265.5 |
| 0.83333333333333304 | 253.5 |
| 0.84375 | 242.5 |
| 0.85416666666666696 | 228.0 |
| 0.86458333333333304 | 213.5 |
| 0.875 | 198.0 |
| 0.88541666666666696 | 184.0 |
| 0.89583333333333304 | 175.0 |
| 0.90625 | 166.5 |
| 0.91666666666666696 | 158.0 |
| 0.92708333333333304 | 151.5 |
| 0.9375 | 142.5 |
| 0.94791666666666696 | 133.5 |
| 0.95833333333333304 | 129.0 |
| 0.96875 | 128.0 |
| 0.97916666666666696 | 125.5 |
| 0.98958333333333304 | 119.5 |
### Chart
| Category | |
|---|---|
| 0 | 93.0 |
| 1.0416666666666666E-2 | 89.0 |
| 2.0833333333333301E-2 | 90.0 |
| 3.125E-2 | 91.0 |
| 4.1666666666666699E-2 | 87.5 |
| 5.2083333333333301E-2 | 87.0 |
| 6.25E-2 | 88.5 |
| 7.2916666666666699E-2 | 88.0 |
| 8.3333333333333301E-2 | 86.5 |
| 9.375E-2 | 85.5 |
| 0.104166666666667 | 83.0 |
| 0.114583333333333 | 86.0 |
| 0.125 | 90.5 |
| 0.13541666666666699 | 90.0 |
| 0.14583333333333301 | 86.0 |
| 0.15625 | 85.5 |
| 0.16666666666666699 | 88.0 |
| 0.17708333333333301 | 89.0 |
| 0.1875 | 91.5 |
| 0.19791666666666699 | 97.0 |
| 0.20833333333333301 | 99.0 |
| 0.21875 | 97.5 |
| 0.22916666666666699 | 96.0 |
| 0.23958333333333301 | 98.0 |
| 0.25 | 103.0 |
| 0.26041666666666702 | 108.0 |
| 0.27083333333333298 | 115.5 |
| 0.28125 | 119.5 |
| 0.29166666666666702 | 113.5 |
| 0.30208333333333298 | 108.0 |
| 0.3125 | 107.0 |
| 0.32291666666666702 | 106.0 |
| 0.33333333333333298 | 119.0 |
| 0.34375 | 160.0 |
| 0.35416666666666702 | 197.5 |
| 0.36458333333333298 | 207.0 |
| 0.375 | 196.0 |
| 0.38541666666666702 | 179.0 |
| 0.39583333333333298 | 166.5 |
| 0.40625 | 160.0 |
| 0.41666666666666702 | 152.5 |
| 0.42708333333333298 | 141.5 |
| 0.4375 | 130.5 |
| 0.44791666666666702 | 123.0 |
| 0.45833333333333298 | 119.5 |
| 0.46875 | 114.0 |
| 0.47916666666666702 | 108.5 |
| 0.48958333333333298 | 102.5 |
| 0.5 | 109.5 |
| 0.51041666666666696 | 150.5 |
| 0.52083333333333304 | 198.5 |
| 0.53125 | 210.5 |
| 0.54166666666666696 | 192.5 |
| 0.55208333333333304 | 177.0 |
| 0.5625 | 176.5 |
| 0.57291666666666696 | 176.5 |
| 0.58333333333333304 | 173.5 |
| 0.59375 | 168.5 |
| 0.60416666666666696 | 159.0 |
| 0.61458333333333304 | 153.5 |
| 0.625 | 149.5 |
| 0.63541666666666696 | 145.0 |
| 0.64583333333333304 | 141.5 |
| 0.65625 | 136.0 |
| 0.66666666666666696 | 129.0 |
| 0.67708333333333304 | 121.5 |
| 0.6875 | 115.0 |
| 0.69791666666666696 | 112.0 |
| 0.70833333333333304 | 109.5 |
| 0.71875 | 109.5 |
| 0.72916666666666696 | 110.0 |
| 0.73958333333333304 | 108.0 |
| 0.75 | 115.5 |
| 0.76041666666666696 | 151.5 |
| 0.77083333333333304 | 200.5 |
| 0.78125 | 240.0 |
| 0.79166666666666696 | 269.0 |
| 0.80208333333333304 | 278.5 |
| 0.8125 | 271.5 |
| 0.82291666666666696 | 259.0 |
| 0.83333333333333304 | 244.5 |
| 0.84375 | 224.0 |
| 0.85416666666666696 | 200.0 |
| 0.86458333333333304 | 179.5 |
| 0.875 | 164.5 |
| 0.88541666666666696 | 158.0 |
| 0.89583333333333304 | 155.0 |
| 0.90625 | 152.5 |
| 0.91666666666666696 | 151.5 |
| 0.92708333333333304 | 146.0 |
| 0.9375 | 140.5 |
| 0.94791666666666696 | 132.5 |
| 0.95833333333333304 | 120.0 |
| 0.96875 | 111.0 |
| 0.97916666666666696 | 104.5 |
| 0.98958333333333304 | 98.5 |Case 11
Case 12
Case 10
(mg/dL)
(mg/dL)
(mg/dL)
(mg/dL)
(mg/dL)
(mg/dL)
### Chart
| Category | |
|---|---|
| 0 | 147.0 |
| 1.0416666666666666E-2 | 138.0 |
| 2.0833333333333301E-2 | 136.0 |
| 3.125E-2 | 136.5 |
| 4.1666666666666699E-2 | 134.5 |
| 5.2083333333333301E-2 | 134.0 |
| 6.25E-2 | 131.5 |
| 7.2916666666666699E-2 | 122.5 |
| 8.3333333333333301E-2 | 111.5 |
| 9.375E-2 | 104.5 |
| 0.104166666666667 | 100.0 |
| 0.114583333333333 | 94.5 |
| 0.125 | 96.5 |
| 0.13541666666666699 | 103.0 |
| 0.14583333333333301 | 109.0 |
| 0.15625 | 110.5 |
| 0.16666666666666699 | 107.0 |
| 0.17708333333333301 | 108.5 |
| 0.1875 | 108.5 |
| 0.19791666666666699 | 104.5 |
| 0.20833333333333301 | 103.0 |
| 0.21875 | 101.0 |
| 0.22916666666666699 | 98.0 |
| 0.23958333333333301 | 96.5 |
| 0.25 | 95.5 |
| 0.26041666666666702 | 97.0 |
| 0.27083333333333298 | 101.5 |
| 0.28125 | 100.5 |
| 0.29166666666666702 | 94.0 |
| 0.30208333333333298 | 91.0 |
| 0.3125 | 93.0 |
| 0.32291666666666702 | 95.0 |
| 0.33333333333333298 | 100.5 |
| 0.34375 | 137.0 |
| 0.35416666666666702 | 196.5 |
| 0.36458333333333298 | 230.5 |
| 0.375 | 240.0 |
| 0.38541666666666702 | 234.5 |
| 0.39583333333333298 | 228.5 |
| 0.40625 | 221.5 |
| 0.41666666666666702 | 207.5 |
| 0.42708333333333298 | 194.0 |
| 0.4375 | 188.5 |
| 0.44791666666666702 | 185.0 |
| 0.45833333333333298 | 176.0 |
| 0.46875 | 168.5 |
| 0.47916666666666702 | 166.5 |
| 0.48958333333333298 | 168.5 |
| 0.5 | 184.5 |
| 0.51041666666666696 | 216.5 |
| 0.52083333333333304 | 240.5 |
| 0.53125 | 262.5 |
| 0.54166666666666696 | 279.0 |
| 0.55208333333333304 | 287.0 |
| 0.5625 | 289.0 |
| 0.57291666666666696 | 277.5 |
| 0.58333333333333304 | 254.5 |
| 0.59375 | 237.0 |
| 0.60416666666666696 | 228.5 |
| 0.61458333333333304 | 221.0 |
| 0.625 | 218.0 |
| 0.63541666666666696 | 225.5 |
| 0.64583333333333304 | 230.5 |
| 0.65625 | 226.0 |
| 0.66666666666666696 | 229.0 |
| 0.67708333333333304 | 236.0 |
| 0.6875 | 234.5 |
| 0.69791666666666696 | 231.0 |
| 0.70833333333333304 | 232.5 |
| 0.71875 | 235.0 |
| 0.72916666666666696 | 235.5 |
| 0.73958333333333304 | 235.5 |
| 0.75 | 240.5 |
| 0.76041666666666696 | 258.0 |
| 0.77083333333333304 | 287.5 |
| 0.78125 | 315.0 |
| 0.79166666666666696 | 325.0 |
| 0.80208333333333304 | 322.5 |
| 0.8125 | 321.5 |
| 0.82291666666666696 | 318.5 |
| 0.83333333333333304 | 313.5 |
| 0.84375 | 298.5 |
| 0.85416666666666696 | 289.5 |
| 0.86458333333333304 | 286.0 |
| 0.875 | 268.0 |
| 0.88541666666666696 | 247.0 |
| 0.89583333333333304 | 235.5 |
| 0.90625 | 232.0 |
| 0.91666666666666696 | 225.0 |
| 0.92708333333333304 | 208.0 |
| 0.9375 | 189.5 |
| 0.94791666666666696 | 184.5 |
| 0.95833333333333304 | 181.0 |
| 0.96875 | 170.0 |
| 0.97916666666666696 | 161.0 |
| 0.98958333333333304 | 156.0 |
### Chart
| Category | |
|---|---|
| 0 | 123.0 |
| 1.0416666666666666E-2 | 118.5 |
| 2.0833333333333301E-2 | 115.0 |
| 3.125E-2 | 110.0 |
| 4.1666666666666699E-2 | 104.0 |
| 5.2083333333333301E-2 | 99.0 |
| 6.25E-2 | 95.0 |
| 7.2916666666666699E-2 | 94.0 |
| 8.3333333333333301E-2 | 96.0 |
| 9.375E-2 | 97.5 |
| 0.104166666666667 | 99.0 |
| 0.114583333333333 | 101.5 |
| 0.125 | 99.0 |
| 0.13541666666666699 | 94.5 |
| 0.14583333333333301 | 91.5 |
| 0.15625 | 87.5 |
| 0.16666666666666699 | 85.0 |
| 0.17708333333333301 | 83.5 |
| 0.1875 | 83.0 |
| 0.19791666666666699 | 83.5 |
| 0.20833333333333301 | 84.0 |
| 0.21875 | 82.0 |
| 0.22916666666666699 | 81.0 |
| 0.23958333333333301 | 80.0 |
| 0.25 | 79.5 |
| 0.26041666666666702 | 79.5 |
| 0.27083333333333298 | 80.5 |
| 0.28125 | 79.0 |
| 0.29166666666666702 | 75.0 |
| 0.30208333333333298 | 73.5 |
| 0.3125 | 73.5 |
| 0.32291666666666702 | 75.0 |
| 0.33333333333333298 | 81.0 |
| 0.34375 | 105.0 |
| 0.35416666666666702 | 152.0 |
| 0.36458333333333298 | 185.0 |
| 0.375 | 183.5 |
| 0.38541666666666702 | 175.0 |
| 0.39583333333333298 | 168.0 |
| 0.40625 | 162.0 |
| 0.41666666666666702 | 151.5 |
| 0.42708333333333298 | 135.5 |
| 0.4375 | 127.0 |
| 0.44791666666666702 | 125.0 |
| 0.45833333333333298 | 126.0 |
| 0.46875 | 126.5 |
| 0.47916666666666702 | 127.0 |
| 0.48958333333333298 | 126.5 |
| 0.5 | 131.0 |
| 0.51041666666666696 | 138.5 |
| 0.52083333333333304 | 160.0 |
| 0.53125 | 181.5 |
| 0.54166666666666696 | 188.0 |
| 0.55208333333333304 | 194.5 |
| 0.5625 | 205.5 |
| 0.57291666666666696 | 204.5 |
| 0.58333333333333304 | 195.5 |
| 0.59375 | 187.0 |
| 0.60416666666666696 | 180.5 |
| 0.61458333333333304 | 178.0 |
| 0.625 | 183.5 |
| 0.63541666666666696 | 192.5 |
| 0.64583333333333304 | 198.0 |
| 0.65625 | 196.0 |
| 0.66666666666666696 | 189.5 |
| 0.67708333333333304 | 183.0 |
| 0.6875 | 179.0 |
| 0.69791666666666696 | 178.0 |
| 0.70833333333333304 | 175.0 |
| 0.71875 | 171.0 |
| 0.72916666666666696 | 170.5 |
| 0.73958333333333304 | 170.5 |
| 0.75 | 169.0 |
| 0.76041666666666696 | 174.5 |
| 0.77083333333333304 | 197.5 |
| 0.78125 | 224.0 |
| 0.79166666666666696 | 239.5 |
| 0.80208333333333304 | 246.5 |
| 0.8125 | 243.0 |
| 0.82291666666666696 | 235.0 |
| 0.83333333333333304 | 229.5 |
| 0.84375 | 225.0 |
| 0.85416666666666696 | 220.0 |
| 0.86458333333333304 | 215.0 |
| 0.875 | 203.5 |
| 0.88541666666666696 | 188.0 |
| 0.89583333333333304 | 179.0 |
| 0.90625 | 173.0 |
| 0.91666666666666696 | 171.0 |
| 0.92708333333333304 | 170.0 |
| 0.9375 | 165.5 |
| 0.94791666666666696 | 157.0 |
| 0.95833333333333304 | 149.5 |
| 0.96875 | 143.0 |
| 0.97916666666666696 | 134.5 |
| 0.98958333333333304 | 127.0 |
### Chart
| Category | |
|---|---|
| 0 | 103.5 |
| 1.0416666666666666E-2 | 101.0 |
| 2.0833333333333301E-2 | 97.5 |
| 3.125E-2 | 97.0 |
| 4.1666666666666699E-2 | 96.0 |
| 5.2083333333333301E-2 | 95.5 |
| 6.25E-2 | 96.5 |
| 7.2916666666666699E-2 | 96.5 |
| 8.3333333333333301E-2 | 96.5 |
| 9.375E-2 | 98.5 |
| 0.104166666666667 | 95.5 |
| 0.114583333333333 | 91.5 |
| 0.125 | 86.5 |
| 0.13541666666666699 | 81.0 |
| 0.14583333333333301 | 78.5 |
| 0.15625 | 74.0 |
| 0.16666666666666699 | 70.0 |
| 0.17708333333333301 | 73.0 |
| 0.1875 | 75.0 |
| 0.19791666666666699 | 75.5 |
| 0.20833333333333301 | 78.0 |
| 0.21875 | 80.5 |
| 0.22916666666666699 | 81.0 |
| 0.23958333333333301 | 80.5 |
| 0.25 | 81.0 |
| 0.26041666666666702 | 81.5 |
| 0.27083333333333298 | 80.5 |
| 0.28125 | 79.5 |
| 0.29166666666666702 | 80.5 |
| 0.30208333333333298 | 81.5 |
| 0.3125 | 81.0 |
| 0.32291666666666702 | 79.5 |
| 0.33333333333333298 | 82.0 |
| 0.34375 | 94.0 |
| 0.35416666666666702 | 114.0 |
| 0.36458333333333298 | 129.5 |
| 0.375 | 141.5 |
| 0.38541666666666702 | 148.5 |
| 0.39583333333333298 | 147.0 |
| 0.40625 | 138.0 |
| 0.41666666666666702 | 128.0 |
| 0.42708333333333298 | 122.0 |
| 0.4375 | 114.5 |
| 0.44791666666666702 | 104.5 |
| 0.45833333333333298 | 94.0 |
| 0.46875 | 90.0 |
| 0.47916666666666702 | 95.5 |
| 0.48958333333333298 | 99.5 |
| 0.5 | 105.0 |
| 0.51041666666666696 | 116.5 |
| 0.52083333333333304 | 129.5 |
| 0.53125 | 142.0 |
| 0.54166666666666696 | 153.0 |
| 0.55208333333333304 | 162.0 |
| 0.5625 | 164.0 |
| 0.57291666666666696 | 154.0 |
| 0.58333333333333304 | 140.0 |
| 0.59375 | 133.0 |
| 0.60416666666666696 | 119.5 |
| 0.61458333333333304 | 109.5 |
| 0.625 | 110.0 |
| 0.63541666666666696 | 117.0 |
| 0.64583333333333304 | 124.5 |
| 0.65625 | 128.5 |
| 0.66666666666666696 | 133.0 |
| 0.67708333333333304 | 135.5 |
| 0.6875 | 135.0 |
| 0.69791666666666696 | 137.5 |
| 0.70833333333333304 | 140.5 |
| 0.71875 | 144.5 |
| 0.72916666666666696 | 149.5 |
| 0.73958333333333304 | 157.0 |
| 0.75 | 164.5 |
| 0.76041666666666696 | 137.5 |
| 0.77083333333333304 | 143.5 |
| 0.78125 | 151.0 |
| 0.79166666666666696 | 158.5 |
| 0.80208333333333304 | 169.0 |
| 0.8125 | 175.0 |
| 0.82291666666666696 | 171.0 |
| 0.83333333333333304 | 164.0 |
| 0.84375 | 151.0 |
| 0.85416666666666696 | 135.0 |
| 0.86458333333333304 | 121.0 |
| 0.875 | 105.0 |
| 0.88541666666666696 | 93.5 |
| 0.89583333333333304 | 90.5 |
| 0.90625 | 89.5 |
| 0.91666666666666696 | 88.0 |
| 0.92708333333333304 | 91.0 |
| 0.9375 | 98.5 |
| 0.94791666666666696 | 101.5 |
| 0.95833333333333304 | 98.5 |
| 0.96875 | 96.5 |
| 0.97916666666666696 | 98.5 |
| 0.98958333333333304 | 101.5 |
### Chart
| Category | |
|---|---|
| 0 | 81.0 |
| 1.0416666666666666E-2 | 79.0 |
| 2.0833333333333301E-2 | 79.5 |
| 3.125E-2 | 80.0 |
| 4.1666666666666699E-2 | 80.0 |
| 5.2083333333333301E-2 | 79.0 |
| 6.25E-2 | 77.5 |
| 7.2916666666666699E-2 | 78.5 |
| 8.3333333333333301E-2 | 79.5 |
| 9.375E-2 | 77.5 |
| 0.104166666666667 | 75.5 |
| 0.114583333333333 | 78.0 |
| 0.125 | 79.5 |
| 0.13541666666666699 | 77.0 |
| 0.14583333333333301 | 76.0 |
| 0.15625 | 76.0 |
| 0.16666666666666699 | 71.0 |
| 0.17708333333333301 | 69.5 |
| 0.1875 | 72.0 |
| 0.19791666666666699 | 73.0 |
| 0.20833333333333301 | 73.0 |
| 0.21875 | 71.5 |
| 0.22916666666666699 | 69.0 |
| 0.23958333333333301 | 69.0 |
| 0.25 | 72.5 |
| 0.26041666666666702 | 75.0 |
| 0.27083333333333298 | 76.0 |
| 0.28125 | 78.0 |
| 0.29166666666666702 | 79.0 |
| 0.30208333333333298 | 79.0 |
| 0.3125 | 77.5 |
| 0.32291666666666702 | 77.0 |
| 0.33333333333333298 | 77.5 |
| 0.34375 | 85.5 |
| 0.35416666666666702 | 103.0 |
| 0.36458333333333298 | 114.5 |
| 0.375 | 117.5 |
| 0.38541666666666702 | 118.5 |
| 0.39583333333333298 | 114.5 |
| 0.40625 | 108.5 |
| 0.41666666666666702 | 108.0 |
| 0.42708333333333298 | 109.5 |
| 0.4375 | 107.5 |
| 0.44791666666666702 | 104.0 |
| 0.45833333333333298 | 103.0 |
| 0.46875 | 104.0 |
| 0.47916666666666702 | 104.0 |
| 0.48958333333333298 | 101.0 |
| 0.5 | 102.0 |
| 0.51041666666666696 | 105.0 |
| 0.52083333333333304 | 112.0 |
| 0.53125 | 117.5 |
| 0.54166666666666696 | 118.5 |
| 0.55208333333333304 | 116.5 |
| 0.5625 | 112.5 |
| 0.57291666666666696 | 110.0 |
| 0.58333333333333304 | 111.0 |
| 0.59375 | 114.5 |
| 0.60416666666666696 | 120.0 |
| 0.61458333333333304 | 123.5 |
| 0.625 | 124.5 |
| 0.63541666666666696 | 124.5 |
| 0.64583333333333304 | 123.5 |
| 0.65625 | 120.5 |
| 0.66666666666666696 | 117.0 |
| 0.67708333333333304 | 116.0 |
| 0.6875 | 116.5 |
| 0.69791666666666696 | 115.5 |
| 0.70833333333333304 | 113.5 |
| 0.71875 | 112.5 |
| 0.72916666666666696 | 112.5 |
| 0.73958333333333304 | 113.5 |
| 0.75 | 117.0 |
| 0.76041666666666696 | 125.0 |
| 0.77083333333333304 | 132.0 |
| 0.78125 | 143.5 |
| 0.79166666666666696 | 152.0 |
| 0.80208333333333304 | 155.0 |
| 0.8125 | 154.0 |
| 0.82291666666666696 | 150.5 |
| 0.83333333333333304 | 142.5 |
| 0.84375 | 129.5 |
| 0.85416666666666696 | 116.0 |
| 0.86458333333333304 | 106.5 |
| 0.875 | 102.0 |
| 0.88541666666666696 | 100.5 |
| 0.89583333333333304 | 99.5 |
| 0.90625 | 97.5 |
| 0.91666666666666696 | 94.0 |
| 0.92708333333333304 | 92.5 |
| 0.9375 | 89.5 |
| 0.94791666666666696 | 86.0 |
| 0.95833333333333304 | 82.0 |
| 0.96875 | 80.5 |
| 0.97916666666666696 | 82.5 |
| 0.98958333333333304 | 82.5 |
### Chart
| Category | |
|---|---|
| 0 | 104.0 |
| 1.0416666666666666E-2 | 105.5 |
| 2.0833333333333301E-2 | 111.0 |
| 3.125E-2 | 110.5 |
| 4.1666666666666699E-2 | 107.5 |
| 5.2083333333333301E-2 | 114.0 |
| 6.25E-2 | 118.5 |
| 7.2916666666666699E-2 | 116.5 |
| 8.3333333333333301E-2 | 110.5 |
| 9.375E-2 | 107.0 |
| 0.104166666666667 | 110.0 |
| 0.114583333333333 | 108.0 |
| 0.125 | 105.5 |
| 0.13541666666666699 | 105.0 |
| 0.14583333333333301 | 106.0 |
| 0.15625 | 109.5 |
| 0.16666666666666699 | 110.0 |
| 0.17708333333333301 | 110.0 |
| 0.1875 | 113.5 |
| 0.19791666666666699 | 114.5 |
| 0.20833333333333301 | 111.5 |
| 0.21875 | 109.5 |
| 0.22916666666666699 | 107.5 |
| 0.23958333333333301 | 109.5 |
| 0.25 | 113.0 |
| 0.26041666666666702 | 116.0 |
| 0.27083333333333298 | 118.0 |
| 0.28125 | 117.0 |
| 0.29166666666666702 | 121.0 |
| 0.30208333333333298 | 125.5 |
| 0.3125 | 124.0 |
| 0.32291666666666702 | 123.0 |
| 0.33333333333333298 | 130.0 |
| 0.34375 | 150.0 |
| 0.35416666666666702 | 166.0 |
| 0.36458333333333298 | 167.5 |
| 0.375 | 159.0 |
| 0.38541666666666702 | 145.5 |
| 0.39583333333333298 | 137.0 |
| 0.40625 | 129.5 |
| 0.41666666666666702 | 119.0 |
| 0.42708333333333298 | 111.0 |
| 0.4375 | 105.5 |
| 0.44791666666666702 | 98.0 |
| 0.45833333333333298 | 89.5 |
| 0.46875 | 83.5 |
| 0.47916666666666702 | 80.0 |
| 0.48958333333333298 | 81.0 |
| 0.5 | 88.5 |
| 0.51041666666666696 | 99.5 |
| 0.52083333333333304 | 107.0 |
| 0.53125 | 111.5 |
| 0.54166666666666696 | 112.0 |
| 0.55208333333333304 | 109.0 |
| 0.5625 | 104.5 |
| 0.57291666666666696 | 99.5 |
| 0.58333333333333304 | 94.0 |
| 0.59375 | 90.0 |
| 0.60416666666666696 | 92.0 |
| 0.61458333333333304 | 99.0 |
| 0.625 | 106.0 |
| 0.63541666666666696 | 112.5 |
| 0.64583333333333304 | 117.0 |
| 0.65625 | 117.5 |
| 0.66666666666666696 | 116.0 |
| 0.67708333333333304 | 112.5 |
| 0.6875 | 106.0 |
| 0.69791666666666696 | 99.0 |
| 0.70833333333333304 | 94.5 |
| 0.71875 | 93.0 |
| 0.72916666666666696 | 93.0 |
| 0.73958333333333304 | 94.5 |
| 0.75 | 101.5 |
| 0.76041666666666696 | 115.0 |
| 0.77083333333333304 | 131.5 |
| 0.78125 | 142.5 |
| 0.79166666666666696 | 142.5 |
| 0.80208333333333304 | 134.5 |
| 0.8125 | 127.5 |
| 0.82291666666666696 | 125.5 |
| 0.83333333333333304 | 122.0 |
| 0.84375 | 118.5 |
| 0.85416666666666696 | 118.5 |
| 0.86458333333333304 | 117.5 |
| 0.875 | 115.0 |
| 0.88541666666666696 | 112.0 |
| 0.89583333333333304 | 106.5 |
| 0.90625 | 103.5 |
| 0.91666666666666696 | 103.0 |
| 0.92708333333333304 | 100.0 |
| 0.9375 | 99.5 |
| 0.94791666666666696 | 97.0 |
| 0.95833333333333304 | 91.5 |
| 0.96875 | 91.5 |
| 0.97916666666666696 | 98.0 |
| 0.98958333333333304 | 103.0 |
### Chart
| Category | |
|---|---|
| 0 | 92.5 |
| 1.0416666666666666E-2 | 93.0 |
| 2.0833333333333301E-2 | 92.0 |
| 3.125E-2 | 90.5 |
| 4.1666666666666699E-2 | 90.0 |
| 5.2083333333333301E-2 | 90.0 |
| 6.25E-2 | 89.5 |
| 7.2916666666666699E-2 | 87.5 |
| 8.3333333333333301E-2 | 88.5 |
| 9.375E-2 | 89.5 |
| 0.104166666666667 | 89.5 |
| 0.114583333333333 | 89.0 |
| 0.125 | 84.5 |
| 0.13541666666666699 | 80.5 |
| 0.14583333333333301 | 88.5 |
| 0.15625 | 98.5 |
| 0.16666666666666699 | 102.5 |
| 0.17708333333333301 | 101.5 |
| 0.1875 | 98.5 |
| 0.19791666666666699 | 95.0 |
| 0.20833333333333301 | 93.5 |
| 0.21875 | 95.5 |
| 0.22916666666666699 | 95.5 |
| 0.23958333333333301 | 95.0 |
| 0.25 | 97.5 |
| 0.26041666666666702 | 101.0 |
| 0.27083333333333298 | 103.5 |
| 0.28125 | 104.5 |
| 0.29166666666666702 | 103.0 |
| 0.30208333333333298 | 103.5 |
| 0.3125 | 105.5 |
| 0.32291666666666702 | 107.0 |
| 0.33333333333333298 | 110.0 |
| 0.34375 | 119.0 |
| 0.35416666666666702 | 129.5 |
| 0.36458333333333298 | 136.5 |
| 0.375 | 138.0 |
| 0.38541666666666702 | 136.0 |
| 0.39583333333333298 | 129.5 |
| 0.40625 | 119.0 |
| 0.41666666666666702 | 109.0 |
| 0.42708333333333298 | 97.0 |
| 0.4375 | 85.5 |
| 0.44791666666666702 | 80.0 |
| 0.45833333333333298 | 77.5 |
| 0.46875 | 74.0 |
| 0.47916666666666702 | 72.0 |
| 0.48958333333333298 | 71.0 |
| 0.5 | 74.0 |
| 0.51041666666666696 | 82.5 |
| 0.52083333333333304 | 89.5 |
| 0.53125 | 94.0 |
| 0.54166666666666696 | 96.0 |
| 0.55208333333333304 | 93.5 |
| 0.5625 | 90.0 |
| 0.57291666666666696 | 90.0 |
| 0.58333333333333304 | 92.0 |
| 0.59375 | 97.0 |
| 0.60416666666666696 | 99.0 |
| 0.61458333333333304 | 98.0 |
| 0.625 | 95.0 |
| 0.63541666666666696 | 94.5 |
| 0.64583333333333304 | 99.0 |
| 0.65625 | 104.0 |
| 0.66666666666666696 | 103.5 |
| 0.67708333333333304 | 96.5 |
| 0.6875 | 89.5 |
| 0.69791666666666696 | 87.0 |
| 0.70833333333333304 | 87.0 |
| 0.71875 | 88.5 |
| 0.72916666666666696 | 88.0 |
| 0.73958333333333304 | 87.0 |
| 0.75 | 90.5 |
| 0.76041666666666696 | 94.0 |
| 0.77083333333333304 | 100.0 |
| 0.78125 | 112.0 |
| 0.79166666666666696 | 124.0 |
| 0.80208333333333304 | 125.0 |
| 0.8125 | 121.5 |
| 0.82291666666666696 | 119.0 |
| 0.83333333333333304 | 118.0 |
| 0.84375 | 115.0 |
| 0.85416666666666696 | 110.5 |
| 0.86458333333333304 | 104.5 |
| 0.875 | 100.5 |
| 0.88541666666666696 | 101.5 |
| 0.89583333333333304 | 100.0 |
| 0.90625 | 95.5 |
| 0.91666666666666696 | 93.5 |
| 0.92708333333333304 | 93.5 |
| 0.9375 | 92.5 |
| 0.94791666666666696 | 92.5 |
| 0.95833333333333304 | 93.5 |
| 0.96875 | 93.0 |
| 0.97916666666666696 | 91.0 |
| 0.98958333333333304 | 91.0 |
