# Supplementary material for: Switching from Insulin Degludec plus Dipeptidyl Peptidase-4 Inhibitor to Insulin Degludec/Liraglutide Improves Glycemic Variability in Patients with Type 2 Diabetes: A Preliminary Prospective Observation Study
Source: J Diabetes Res. 2022 Jan 19;2022:5603864. doi: 10.1155/2022/5603864 (PMC8793345; doi:10.1155/2022/5603864)
Supplement: Supplementary 5 — Supplementary Figure 4: self-measured glucose concentrations during each phase of the day. Mean self-measured glucose concentrations during each phase of the day. The data are for all 12 participants, and the error bars represent the standard deviation of the measurements. All the datasets were normally distributed and analyzed using Student's t-test. The mean preprandial and after-supper glucose concentrations are shown. Black diamond: insulin degludec plus a dipeptidyl peptidase-4 inhibitor; black circle: insulin degludec/liraglutide. ∗P < 0.05; NS: not significant. [file 5603864.f5.pptx]

## Slide 1
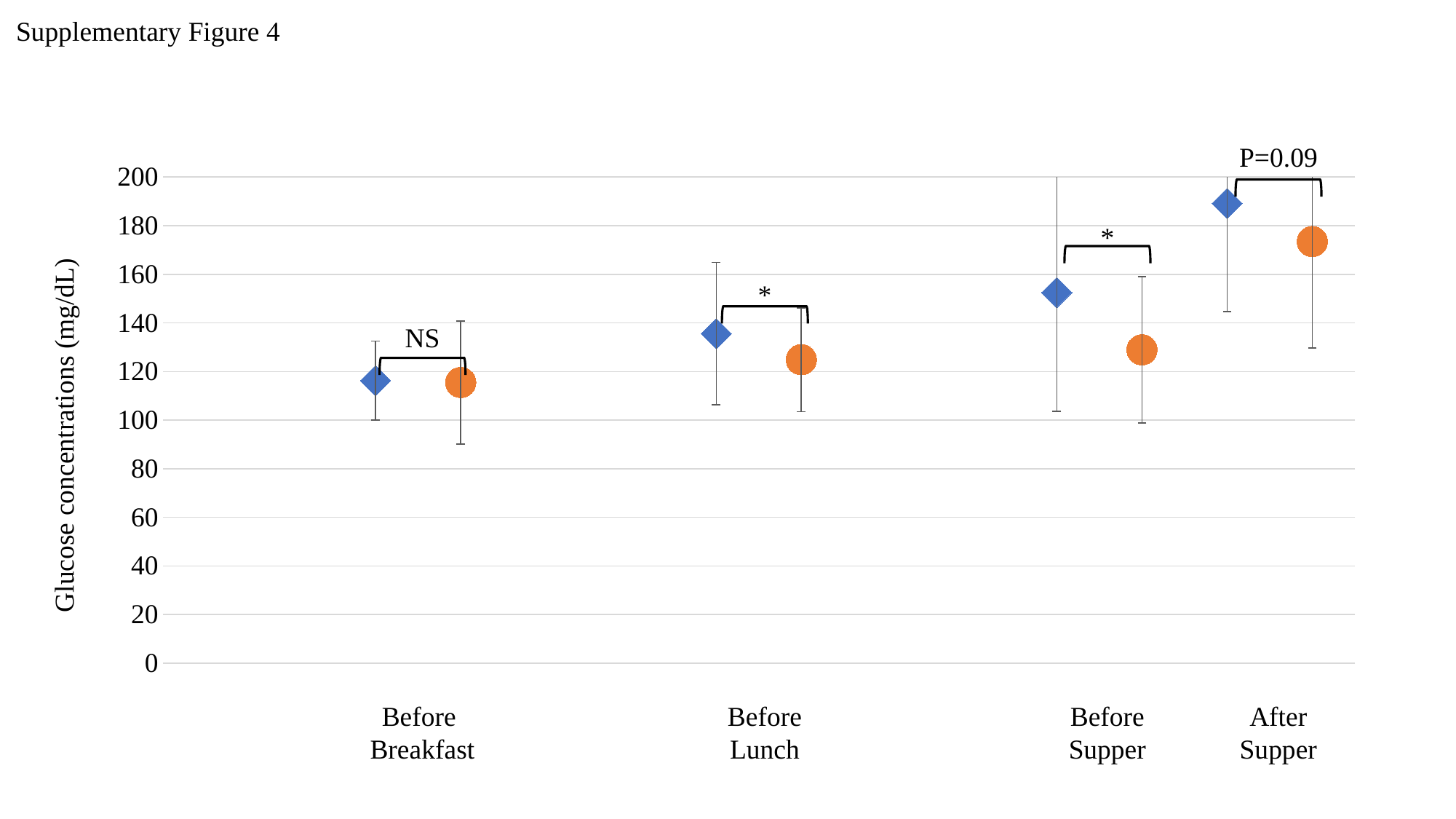

Supplementary Figure 4
P=0.09
### Chart
| Category | 前 | 後 |
|---|---|---|
| 3-6時 | None | None |
| | None | None |
| 朝食時 | 116.25 | None |
| | None | 115.5 |
| 10-12時 | None | None |
| | None | None |
| 昼食時 | 135.58333333333334 | None |
| | None | 124.875 |
| 14-18時 | None | None |
| | None | None |
| 夕食時 | 152.5 | None |
| | None | 128.91666666666666 |
| 21-0時 | 189.125 | None |
*
*
NS
Glucose concentrations (mg/dL)
Midnight
Before
Breakfast
After Breakfast
Before
Lunch
After
Lunch
Before
Supper
After
Supper
